# Bent and Twisted: Synthesis of an Alkoxy-Substituted (1,5)Naphthalene-paracyclophanediene

**DOI:** 10.1021/acs.joc.3c00880

**Published:** 2023-08-30

**Authors:** Arielle Mann, Matthew D. Hannigan, Bianca L. Dumlao, Chunhua T. Hu, Marcus Weck

**Affiliations:** Department of Chemistry and Molecular Design Institute, New York University, New York, New York 10003, United States

## Abstract

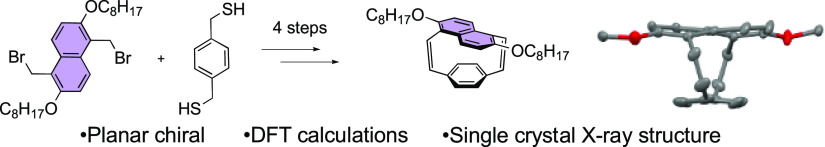

This contribution describes the synthesis of [2.2](1,5)naphthalenoparacyclophane-1,13-diene in four steps from 1,5-bis(bromomethyl)naphthalene and 1,4-benzenedimethanethiol. Consisting of 2,6-dioctyloxynaphthalene and benzene moieties, the effects of differing arene size on the structure, strain energy, and chemical reactivity of the cyclophanediene are examined. Despite a strain energy of 24.3 kcal/mol, the naphthalenoparacyclophanediene was unreactive toward a library of olefin metathesis catalysts. This diminished reactivity can be explained by the steric hindrance of the twisted olefin. Incorporation of an electron donor (naphthalene) into the rigid paracyclophanediene structure can allow for applications in optoelectronics, chiral ligands, and planar chiral materials.

## Introduction

**Figure 1:**
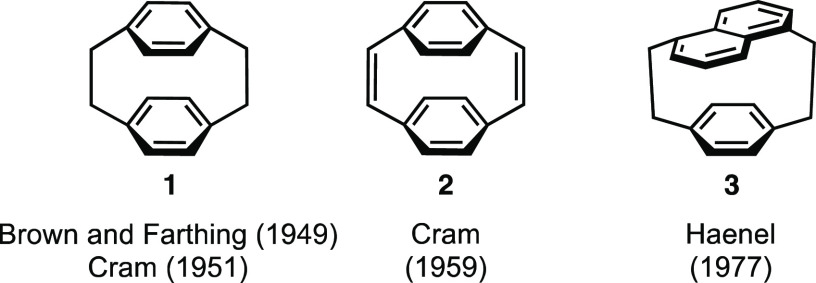
Structures of [2.2]paracyclophane (pCp) **1**,^19,20^ [2.2]paracyclophane-1,9-dienes (pCpd) **2**,^21^ and [2.2](1,5)naphthalenoparacyclophane **3**.^22^

New classes of strained aromatic molecules are commonly pursued by chemists because these “bent and battered”^1^ scaffolds offer insights into the nature of bonding and often yield surprising charge transport, electronic, and optical properties.^2−4^ A class of strained molecules receiving particular attention are cyclophanes, which are synthetically challenging compounds featuring nonplanar aromatic systems. The structure of cyclophanes can be broken into two components: the aromatic “decks” and their connecting carbon “bridges”.^5^ [2.2]Paracyclophane (pCp) **1** (Figure 1), the prototypical cyclophane, with *para*-bridged benzene moieties as decks and ethylenes as bridges, has long been investigated, and its chemistry is well-understood, enabling access to a variety of ring substitutions (especially on the decks).^2^ Many applications have been found for pCp such as ligands for chiral catalysts used in stereoselective synthesis,^6,7^ chiroptics and optoelectronics,^5,8−10^ pharmaceuticals,^11,12^ and materials science.^7,13−15^ The much more strained structural analog of **1** is [2.2]paracyclophane-1,9-diene (pCpd) **2**,^16^ which has ethynylene bridges instead of ethylene bridges, bringing the benzene decks closer together than in the parent compound **1** resulting in more-bent benzenes.^17^ While altering the aromatic decks of pCp is common, dienes like pCpd have seen much less structural diversity due to the harsher reaction conditions required for their synthesis.^18^

Compounds related to pCp and pCpd are [2.2]naphthalenophane and [2.2]naphthalenophane-1,13-diene, where both benzene moieties are replaced with naphthalene, which is typically substituted at the (1,4),^23^ (1,5),^24^ (2,6),^25,26^ or (2,7)^27^ positions. Mixing the arenes affords [2.2]naphthalenoparacyclophanes (such as **3**), a highly strained class of cyclophane that has been studied by Haenel and Reiss.^22,28,29^ These compounds are distinct from pCps due to the incorporation of an electron donor to the cyclophane scaffold and (when not substituted at the 1,4 positions) feature a mismatch in size of the arenes being bridged causing unique torsional and transannular interactions.^29−31^

In this contribution, we report the synthesis of dioctyloxy-[2.2](1,5)naphthalenoparacyclophane-1,13-diene (**9**). The impact of the differing arene sizes on the ring strain and chemical reactivity of **9** is examined and compared to the structurally similar pCpd. We propose that expanding the paracyclophanediene scaffold to include an electron-rich naphthalene moiety could provide unique interactions to tune structural, through space, and optoelectronic properties.^4^ In addition, these scaffolds offer possible applications in polymer chemistry as monomers for ring-opening metathesis polymerization (ROMP) to afford poly(1,5-naphthylene-*co*-*p*-phenylenevinylene), a conjugated donor-containing polymer.

## Results and Discussion

**Scheme 1:**
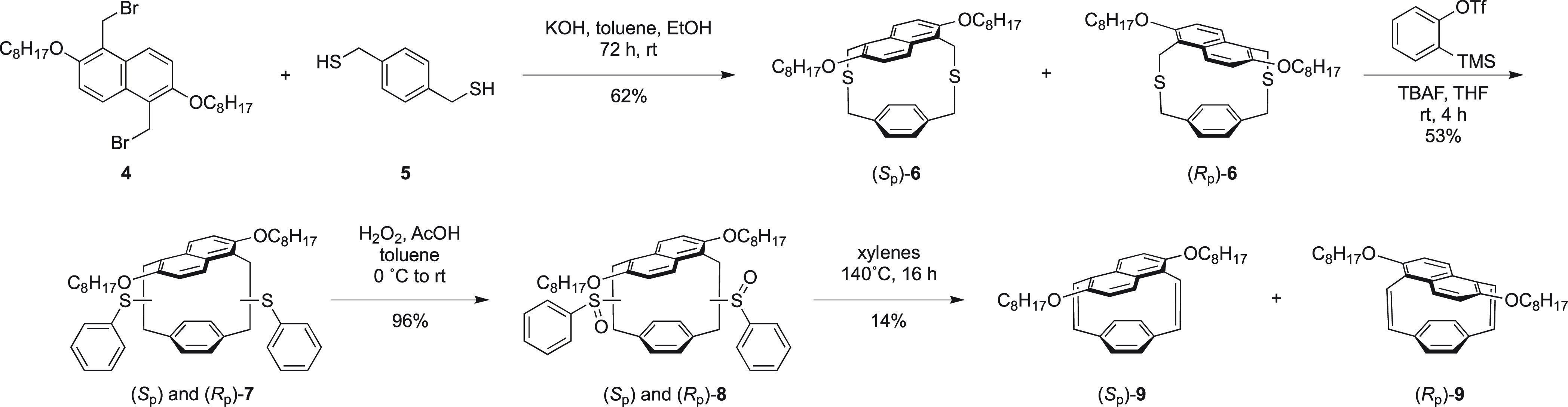
Synthesis of 2,6-Dioctyloxy-[2.2](1,5)naphthalenoparacyclophane-1,13-diene (**9**)

To synthesize **9**, the common synthetic route toward pCpd was followed.^18^ 1,5-Bis(bromomethyl)-2,6-bis(octyloxy)naphthalene **4**, which was easily synthesized using a Blanc-like bromomethylation,^32^ was combined with dithiol **5** over 3 days in dilute conditions to afford dithia[3.3](1,5)naphthalenoparacyclophane **6**. The structure of this compound was confirmed using single-crystal X-ray diffraction (XRD) with a racemic crystal grown from slow evaporation from acetone at room temperature (Figure S16). A benzyne-induced Stevens rearrangement successfully ring-contracted the paracyclophane to the bis(sulfide) [2.2]naphthalenoparacyclophane **7**. Upon oxidation using hydrogen peroxide in acetic acid to afford bis(sulfoxide) **8**, the thermal pyrolysis-induced elimination successfully afforded **9** (Scheme 1). Substituted naphthalenoparacyclophanes are expected to form planar chiral enantiomers.^7,33^ Using chiral HPLC, compounds **6** and **9** were confirmed to be racemic mixtures of the *S*_p_ and *R*_p_ enantiomers (SI Section 2).

**Figure 2:**
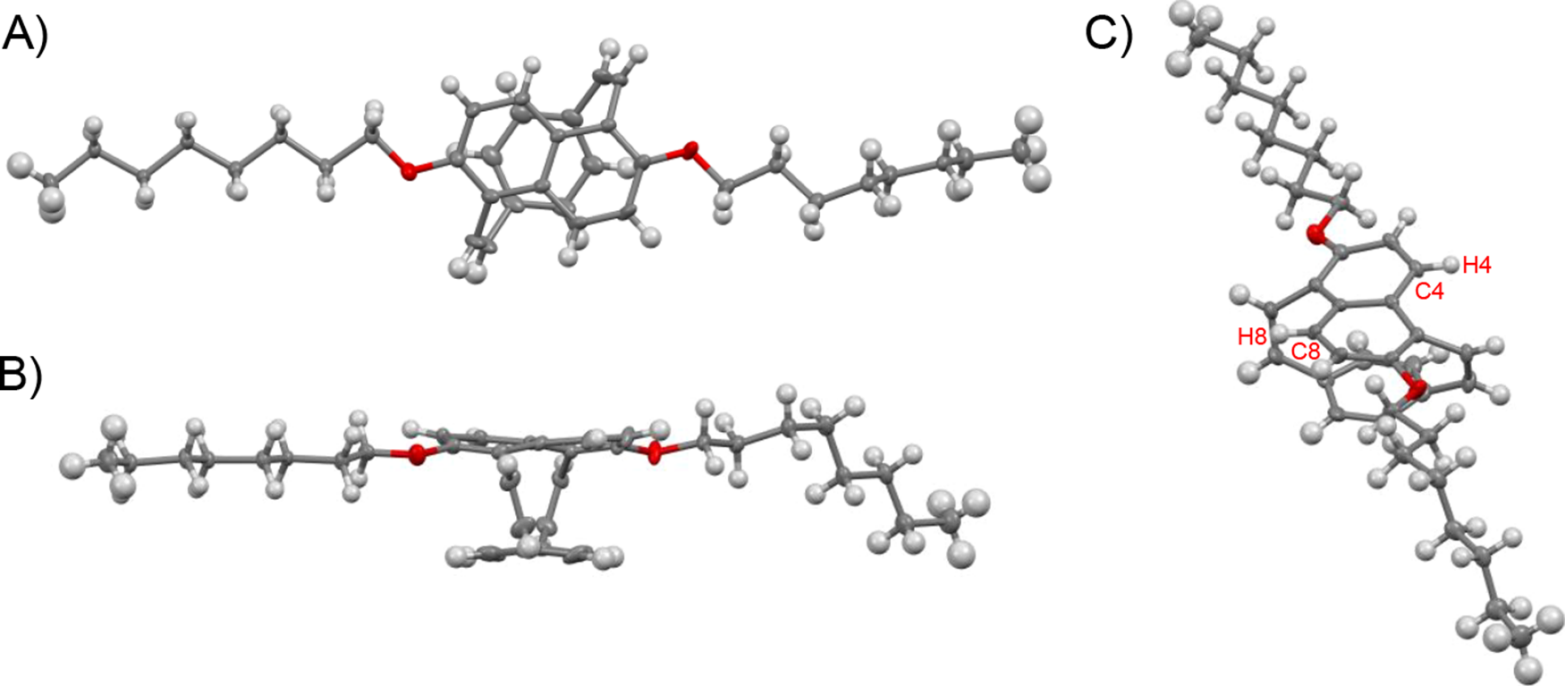
(A–C) Three views of the single-crystal X-ray structure of [2.2]naphthalenoparacyclophanediene **9** with thermal ellipsoids shown at 50% probability.

The structure of **9** was confirmed by single-crystal XRD using a crystal grown by vapor diffusion of hexanes into a solution of the racemic mixture of **9** in dichloromethane at −10 °C. The bridged naphthalene and benzene are centered over each other displaying a face-to-face orientation with a distance from the centroid of the benzene to the centroid of the fused naphthalene rings of 2.96 Å (Figure 2A), much closer than the free heterodimer face-to-face distance of 3.70 Å.^34^ This centroid-to-centroid distance of naphthalenoparacyclophanediene **9** is slightly smaller compared to that of [2.2]paracyclophane **1** (3.09 Å)^35^ and **2** (3.14 Å).^17^ Remarkably, unlike **1** and **2**, the bridges of **9** are not parallel to each other and not perpendicular to the plane of the decks. The twisted olefin configurations accommodate the differing arene lengths of the naphthalene (3.73 Å) and the benzene (2.80 Å) by adopting torsion angles of 30.5° between the two bridges. This torsion also causes the dihedral angle between the decks and the bridges to be 65° (Figure 2B), likely enabling more π-conjugation between the alkenes and the arenes compared to **2** (SI Section 7.3).

Both arene decks in **9** distort to nonplanar conformations due to the closer than favorable face-to-face orientation of the arenes and the bridging strain. The benzene moiety adopts a boat-like conformation, while the naphthalene moiety displays a twisted configuration, a common phenomenon observed in acenes under strain.^26,36^ The twisting of the naphthalene is measured by its torsion angle of −19.2° around its central bond. Another way to quantify the distortion of the arenes from planarity in **9** is by measuring the cyclophane’s angles of α and β (Figure S18).^17,35^ Interestingly, the benzene bridgeheads have a sum of α and β about equal to that of the bent-benzenes in **1** but with the naphthalene β value higher than that observed in **2**. The sum for the octyloxynaphthalene bridgeheads is 2.7° larger than that of the benzene deck indicating that the naphthalene moiety is more distorted than the benzene moiety in **9**. Examining α and β individually, **9** demonstrates smaller angles for α meaning the arenes are more planar compared to **1** and **2**. The majority of the bending can be observed from the *ipso* position of the arene to the carbon of the bridgehead, β (Table 1).

**Table 1:** Experimentally Determined Values of α and β

Cyclophane	α (°)	β (°)	α + β (°)
**9**^a^ - naphthalene	10.9	15.5	26.4
**9**^a^ - benzene	10.6	13.1	23.7
**1**(35)	12.6	11.2	23.8
**2**(17)	13	15	28

a Average values from the two olefins (SI Section 9).

Density functional theory (DFT) computations were performed for a truncated version of **9** to examine the effect of structure on the total strain energy of the paracyclophanediene (for a discussion of methods, see SI Section 6). The strain energy, Δ*G*_strain_, of **9** was computed to be 24.3 kcal/mol, significantly lower than that of **2** (39.4 kcal/mol) when computed by the same method.^18^ To elucidate the structural features that contribute to a lower strain energy, an energy decomposition of the total strain energy was performed, similar to the method described by Grimme for paracyclophane strain energies.^37^ From the energy decomposition analysis, it was found that the strain energy in **9** is smaller primarily due to the torsional angle between the arenes and alkenes in the cyclophane which stabilize **9** by increasing conjugation (Scheme S7 and Table S4). Additionally, the substituents play a role in reducing the strain energy: the alkoxy groups stabilize the cyclophane via donor–acceptor interactions between the arenes. The 1,5-substitution pattern of the naphthalene also contributes in reducing the ring strain by destabilizing the ring-opening due to torsional strain present in the ring-opened products (Scheme S10). Although the mismatch in size between the arenes does play a role in adding to the strain of **9 (**Scheme S9), the effect is small compared to the stabilization caused by arene/olefin conjugation and substitution effects (Table S12).

**Scheme 2:**
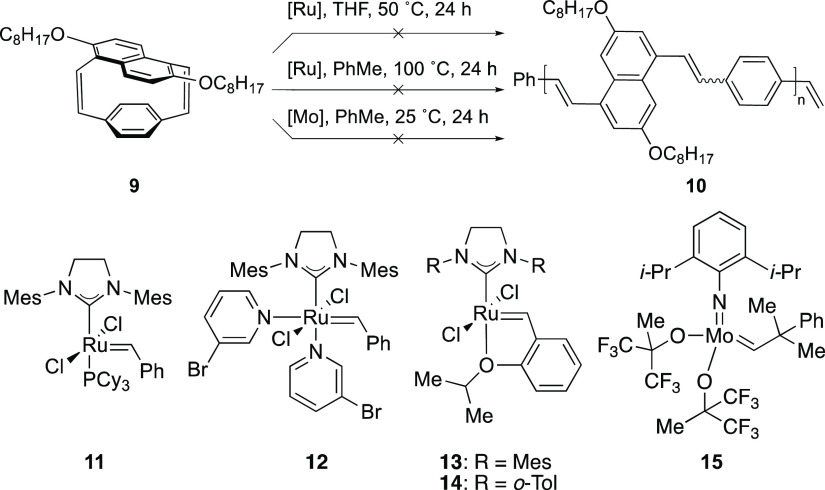
Attempted Ring-Opening Reactions of [2.2]Naphthalenoparacyclophane **9** with Five Different Olefin Metathesis Catalysts For more details of the screened reaction conditions, see SI Section 4.

pCpds have gained popularity as monomers for the living ROMP to afford poly(*p*-phenylene vinylene)s (PPV),^18^ a conjugated polymer that has substantially contributed to the fundamental science of organic semiconductors.^38^ We hypothesized that **9** would be an ideal monomer to afford poly(1,5-naphthylenevinylene-*co*-*p*-phenylenevinylene) **10**, a previously reported polymer that is hypsochromically shifted compared to PPV,^39,40^ in a living manner due to the high ring strain and *n*-octyloxy side-chains necessary for solubilizing the conjugated polymer. Despite trying different versions of ruthenium-based olefin metathesis catalysts (**11**–**14**) at 50 °C in tetrahydrofuran or 100 °C in toluene (common conditions for the polymerizations of olefin-containing cyclophanes),^18^ no reactions were observed (SI Section 4). Further polymerization attempts were also performed with Schrock’s molybdenum catalyst **15** due to its ability to polymerize more sterically encumbered species,^41^ but to no avail (Scheme 2).

**Figure 3:**
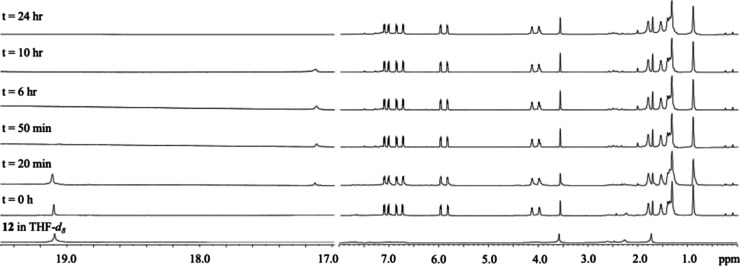
In situ ^1^H NMR spectroscopy experiment of **9** with Grubbs’ third-generation catalyst **12** at 50 °C in THF-*d*_8_.

To further probe the reactivity of **9** with olefin metathesis catalysts, in situ ^1^H NMR spectroscopy experiments were performed. Monitoring the reaction of **9** with Grubbs’ third-generation catalyst **12** at 50 °C, the catalyst is seen to fully initiate with the carbene signal shifting from 19.1 to 17.1 ppm within the first 50 min. After this point, the carbene signal slowly decreases in intensity until it no longer appears, indicating that the catalyst has decomposed at the elevated temperatures over 24 h with no change to the monomer (Figure 3). Alkoxy-substituted pCpds are always ring-opened at elevated temperatures,^18^ and the Ru catalysts are found to be stabilized by coordinating to the aryl ether upon ring opening.^42^ The observed decomposition of Grubbs’ initiator **12** supports that there is no reaction with **9**. This in situ experiment was also repeated with Schrock’s molybdenum catalyst **15** at room temperature and no changes were observed (Figures S8 and S9). When the sample was heated to 40 °C, catalyst **15** was seen to rapidly decompose.

Although the ring strain is reduced in **9**, it is still greater than other olefin-containing paracyclophanes that reacted under similar conditions.^18,43^ Additionally, [2.2]naphthalenophane-1,13-diene with dialkoxy-substituted naphthalene decks react with Grubbs’ catalyst as demonstrated by Yu and coworkers’ investigations into (2,6)[2.2]naphthalenophanediene.^26,44^ Given this, the reactivity of **9** is not expected to be significantly impeded by its lower ring strain or incorporation of a dialkoxynaphthalene deck. We hypothesize, therefore, that this lack of reactivity is due to the steric environment of the olefins. It has been reported for alkoxy-substituted pCpds that the side-chain blocks the face of the olefin closest toward it.^18,45^ For the two open olefin faces of **9**, the Ru–olefin π-complex is unable to form due to the naphthyl carbons C4 and C8 and their corresponding hydrogens sterically blocking the approach of the catalyst to the tilted alkene (Figure 2C). This is additionally supported by DFT calculations showing that these hydrogen atoms cause the Ru–olefin bonds to be significantly enlengthened, weakening the coordination of the ruthenium and preventing entry into the ROMP catalytic cycle (SI Section 7).

## Conclusions

In conclusion, we have synthesized (2,6)-dioctyloxy-[2.2](1,5)naphthalenoparacyclophanediene **9** in four steps. Examining the structure of **9**, the effect of the differing aromatic decks (1,4-benzene and 1,5-naphthalene) can be seen to structurally twist both the cyclophane’s bridges and the naphthalene deck while the benzene deck adopts a boat-like conformation. The ring strain of this small, twisted cyclophane was computed to be 24.3 kcal/mol. Due to the presence of olefin bridges, the ability of **9** to react with olefin metathesis catalysts was examined. It was found to be too sterically hindered from the torsion of the cyclophane and the larger naphthalene deck. While we have demonstrated that highly strained, olefin-containing [2.2]paracyclophanedienes that bear side-chains can be synthesized with more than just benzene moieties, their applications as monomers with olefin metathesis catalysts are not predictable. Further development of ROMP catalysts that can coordinate to very sterically hindered substituted olefins are needed to realize the applications of these new cyclophanes as monomers for olefin metathesis. Investigations of naphthalene-containing pCpd scaffolds can provide unique applications with planar chiral purposes as well as electron-rich conjugated scaffolds to tune structural and optoelectronic properties of materials.

## Experimental Section

All chemicals were purchased from Oakwood Chemicals, TCI Chemicals, Strem, Ambeed, or Millipore-Sigma and used as received unless otherwise indicated. Xylenes were dried via 4 Å molecular sieves prior to usage. All reactions were carried out under ambient conditions unless otherwise noted. Flash column chromatography was performed using silica gel 60 Å (230–400 mesh) from Sorbent Technologies. NMR spectroscopy characterizations were conducted at 25 °C on a Bruker Avance 400 MHz, 500 MHz, or 600 MHz spectrometers. Chemical shifts are reported in ppm and referenced to solvent residual peaks. Splitting patterns are reported as singlet (s), doublet (d), doublet of doublets (dd), triplet (t), quartet (q), and multiplet (m). Structural assignments were made with additional information from gHSQC and gHMBC experiments. Mass spectra of samples in methanol were acquired with an Agilent 6224 Accurate-Mass TOF/LC/MS Spectrometer using an ESI ion source.

Compounds **4**,^32^**5**,^46^ and (trimethylsilyl)phenyl trifluoromethanesulfonate were synthesized according to literature procedures.^47^

### Dithia[3.3]naphthalenoparacyclophane (**6**)

Potassium hydroxide 85% (2.714 g, 42.07 mmol) was dissolved in 700 mL of ethanol in a 2000 mL three-neck round-bottom flask. In a separate flask, compound **4** (6.03 g, 10.57 mmol) and compound **5** (1.79 g, 10.51 mmol) were dissolved in 500 mL of toluene and added into a 500-mL pressure equilibrating addition funnel with an adjustable bore metering plug. Both solutions were degassed using argon for 30 min. The toluene solution was added dropwise into the round-bottom flask over a period of 72 h at room temperature. Once completely added, the reaction mixture was left stirring for an additional 24 h. The solvent was removed using a rotary evaporator. The resulting oil was run through a silica plug and washed copiously with dichloromethane. The excess dichloromethane was extracted with water and brine. The oil was then adhered to silica and purified by flash column chromatography on silica using 3:1–2:1 hexanes:dichloromethane as the eluent. This yielded a clear oil that solidified into a white crystalline solid upon sitting (3.78 g, 62%). ^1^H NMR (500 MHz, CDCl_3_) δ 7.75 (d, *J* = 9.3, 2H), 7.04 (d, *J* = 9.3, 2H), 6.55 (dd, *J* = 7.92, 1.71, 2H), 5.99 (dd, *J* = 7.93, 1.80, 2H), 4.92 (d, *J* = 13.58, 2H), 4.15 (m, 4H), 3.72 (d, *J* = 13.57, 2H), 3.53 (q, *J* = 22.60, 4H), 1.91 (m, 4H), 1.56 (quintet, *J* = 7.39, 4H), 1.384 (m, 20H), 0.91 (t, *J* = 6.89, 6H). ^13^C{^1^H} NMR (125 MHz, CDCl_3_) δ 153.8, 134.8, 128.2, 126.6, 126.0, 125.6, 118.5, 113.9, 69.6, 35.5, 32.0, 29.9, 29.6, 29.5, 26. 5, 25.8, 22.8, 14.3. HRMS (ESI) *m/z* calculated for C_36_H_50_O_2_S_2_Na (M + Na)^+^ 602.3177, found 602.3161.

### Bis(sulfide)-[2.2]naphthalenoparacyclophane (**7**)

Compound **6** (12.00 g, 20.70 mmol) and 2-(trimethylsilyl)phenyl triflate (15.096 mL, 62.20 mmol) were dissolved in 590 mL of tetrahydrofuran in a round-bottom flask. Tetra-*n*-butylammonium fluoride hydrate (TBAF·3H_2_O) (22.89 g, 72.60 mmol) was dissolved in 100 mL of tetrahydrofuran and added to a pressure equilibrating addition funnel with an adjustable bore metering plug. The solution was added dropwise to the round-bottom flask over 4 hours and left stirring overnight. The reaction solution was concentrated in vacuo to a thick, light yellow oil and adhered to silica and purified by flask column chromatography on silica using a gradient eluent of 100:1–2:1 hexanes:dichloromethane. This gave a clear, yellow oil (8.06 g, 53%). HRMS (ESI) *m/z* calculated for C_48_H_58_O_2_S_2_Na (M + Na)^+^ 753.3770, found 753.3798.

### Bis(sulfoxide)-[2.2]naphthalenoparacyclophane (**8**)

Compound **7** (3.04 g, 5.25 mmol) was dissolved in 106 mL of toluene and cooled to 0 °C. Then, 33 mL of acetic acid was added to the reaction mixture followed by 2 mL of hydrogen peroxide (32 wt %) dropwise over a period of 20 min. The reaction was brought to room temperature and allowed to stir overnight. The reaction was then diluted with dichloromethane (150 mL), DI H_2_O (100 mL), and brine (100 mL). The organic layer was extracted, washed with more brine and NaHCO_3(aq)_ three times, and the organic layers were combined, dried over MgSO_4_, and the solvent was removed in vacuo to give a yellow-green oil (3.05 g, 96%). HRMS (ESI) *m/z* calculated for C_48_H_58_O_4_S_2_Na (M + Na)^+^ 785.3669, found 785.3658.

### [2.2]Naphthalenoparacyclophane-1,13-diene (**9**)

Compound **8** (3.05 g, 4.00 mmol) was dissolved in 138.78 mL of degassed anhydrous xylenes and heated at reflux under argon for 20 h. The reaction was allowed to cool to room temperature, and the xylenes were removed in vacuo to give an oil that was purified by flash column chromatography on silica using a gradient eluent of 4:1–2:1 hexanes:dichloromethane to yield a yellow waxy solid (0.297 g, 14%). ^1^H NMR (500 MHz, CDCl_3_) δ 7.16 (d, *J* = 8.93, 2H), 7.06 (d, *J* = 9.79, 2H), 6.94 (d, *J* = 9.78, 2H), 6.74 (d, *J* = 8.94, 2H), 6.04 (dd, *J* = 3.13, 2H), 5.88 (dd, *J* = 3.02, 2H), 4.17 (m, 2H), 4.01 (m, 2H), 1.82 (m, 4H), 1.51 (m, 4H), 1.37 (m, 16H), 0.90 (t, *J* = 6.87, 6H). ^13^C{^1^H} NMR (125 MHz, CDCl_3_) δ 156.0, 136.8, 136.7, 132.4, 131.2, 127.1, 126.88, 126.87, 121.5, 114.9, 70.4, 32.0, 30.0, 29.6, 29.4, 26.3, 22.8, 14.3. HRMS (ESI) *m/z* calculated for C_36_H_46_O_2_K (M + K)^+^ 549.3129, found 549.3155.

### General Procedure for Reaction Screenings of **9** with Olefin Metathesis Catalysts

In a nitrogen-filled glovebox, a stock solution of the desired initiator (10 mol %) was prepared in anhydrous, degassed THF or toluene. Compound **9** (20 mg) was weighed out into a one-dram vial and brought into the glovebox, dissolved in anhydrous, degassed THF or toluene, and transferred to an oven dried Schlenk tube. An appropriate amount of the catalyst solution (**11**,**12**,**13**,**14**, or **15**) was added for [**9**] = 100 mM. The Schlenk tube was sealed and removed from the glovebox where it was subsequently wrapped in aluminum foil and kept at room temperature or placed in an oil bath at 50 °C or 100 °C and stirred. The reaction was cooled to room temperature, and a large excess of deoxygenated ethyl vinyl ether (0.80 mL) was added and allowed to stir at room temperature for 12 h. The reaction mixture was than concentrated down, allowed to dry under vacuum for 1 hour after which a ^1^H NMR spectrum was recorded. The starting material was recovered by column chromatography.

### In Situ ^1^H NMR Spectroscopy Experiments

Compound **9**, as an inseparable mixture of enantiomers (30 mg, 0.041 mmol), and **12** or **15** were individually weighted out into 1 dram vials and brought into a nitrogen-filled glovebox. **9** and the desired catalyst were separately dissolved in THF-*d*_8_ or toluene-*d*_8_ and combined for a total volume of 0.418 mL ([**9**] = 100 mM) and transferred into a *J*-Young NMR tube that was sealed. The sample was removed from the glovebox, wrapped in aluminum foil, and placed in an ice bath. The sample was removed from the aluminum foil and placed into a 600 MHz NMR set to 25 °C from *t* = 0. For the sample with **12**, the spectrometer was heated to 50 °C. ^1^H NMR spectra were recorded every 5 minutes for the first hour and then every 20 min for 24 h.

## Data Availability

The data underlying this study are available in the published article and its Supporting Information.

## Supporting Information Available

Full experimental details, NMR spectra, optical spectra, computational data, and descriptions of crystallography (PDF)
