# Supplementary material for: Bent and Twisted: Synthesis of an Alkoxy-Substituted (1,5)Naphthalene-paracyclophanediene
Source: J Org Chem. 2023 Aug 30;88(18):12971–7. doi: 10.1021/acs.joc.3c00880 (PMC10507662; doi:10.1021/acs.joc.3c00880)

<u>Supporting Information</u>

Bent and Twisted: Synthesis of an Alkoxy-Substituted (1,5)Naphthalene-Paracyclophanediene

Arielle Mann, Matthew D. Hannigan, Bianca L. Dumlao, Chunhua T. Hu, Marcus Weck*

Molecular Design Institute and Department of Chemistry, New York University
New York, NY 10003, USA

*To whom correspondence should be addressed: marcus.weck@nyu.edu

Table of Contents

1. Materials and Methods......S2

2. Chiral HPLC......S3

3. Photophysical Characterization......S4

4. Screening Reactions With Olefin Metathesis Catalysts......S6

5. In situ ^{1}H NMR experiments......S7

6. Strain Energy Calculations......S11

7. Calculations with Ruthenium Catalysts......S20

8. X-Ray Crystallographic Data......S22

9. Determining the angle of α and β......S28

10. XYZ coordinates of Computed Structures......S29

12. References......S48

13. NMR Spectra......S48

1. Materials and Methods

All chemicals were purchased from Oakwood Chemicals, TCI Chemicals, Strem, Ambeed, or Millipore Sigma and used as received unless otherwise indicated. Xylenes was dried via 4 Å molecular sieves prior to usage. All reactions were carried out under ambient conditions unless otherwise noted. Flash column chromatography was performed using silica gel 60 Å (230-400 mesh) from Sorbent Technologies.

NMR spectroscopy characterizations were conducted at 25 ˚C on a Bruker Avance 400 MHz, 500 MHz, or 600 MHz spectrometers. Chemical shifts are reported in ppm and referenced to solvent residual peaks. Splitting patterns are reported as singlet (s), doublet (d), doublet of doublets (dd), triplet (t), quartet (q) and multiplet (m).

Mass spectra of samples in methanol were acquired with an Agilent 6224 Accurate-Mass TOF/LC/MS Spectrometer using an ESI ion-source.

For Chiral HPLC, the samples were separated and analyzed by an Agilent 1260 Infinity HPLC equipped with CHIRALPAK IA-3 column or OD-H01 column.

Absorption spectra were obtained using a Cary 100 UV-VIS Spectrophotometer by Agilent Technologies. Fluorescence spectra were collected on a QuantaMaster 40 Photon Technology International spectrofluorometer equipped with Xenon lamp source, emission and excitation monochromators, excitation correction unit, and PMT detector. All measurements were conducted at 25.0 ± 0.1 °C maintained by a Quantum Northwest cuvette temperature controller. Emission and excitation spectra were corrected for the wavelength-dependent response and wavelength dependent lamp intensity.

2. Chiral HPLC

General procedure: 8 mg of **6** and **9** were dissolved in separate vials in a 1:1 mixture of hexanes and isopropanol.

HPLC conditions for compound **6**: (IA-3 column, 90:10 hexane/iPrOH, 0.700 mL/min)
HPLC conditions for compound **9**: (OD-H01 column, 98:2 hexane/iPrOH, 0.700 mL/min)

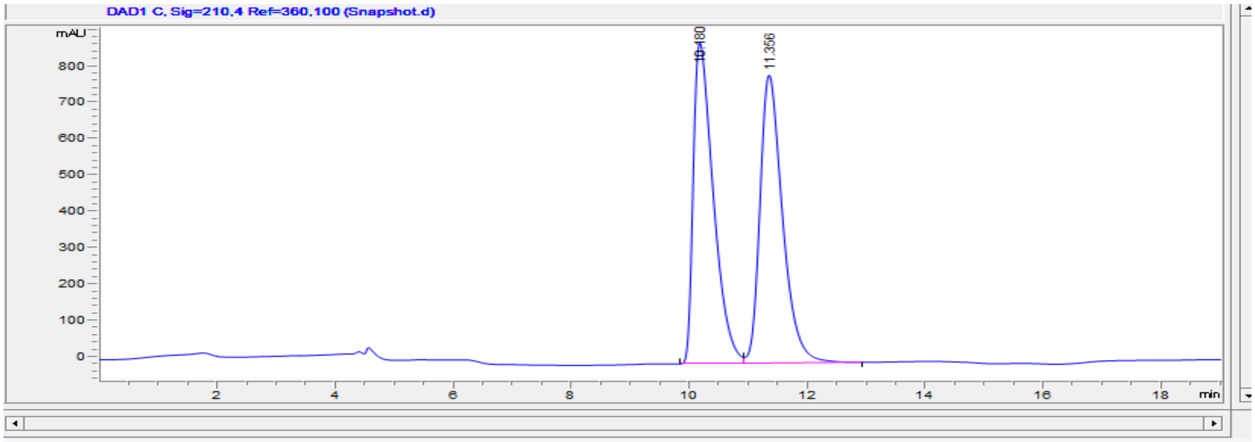

#	Time	Area	Height	Width	Area%	Symmetry
1	10.18	20878.7	881.2	0.3604	49.815	0.452
2	11.356	21033.6	790.8	0.4023	50.185	0.636

Figure S1. Chiral HPLC chromatogram of (S_P)- and (R_P)-**6** showing they form in a 1:1 ratio.

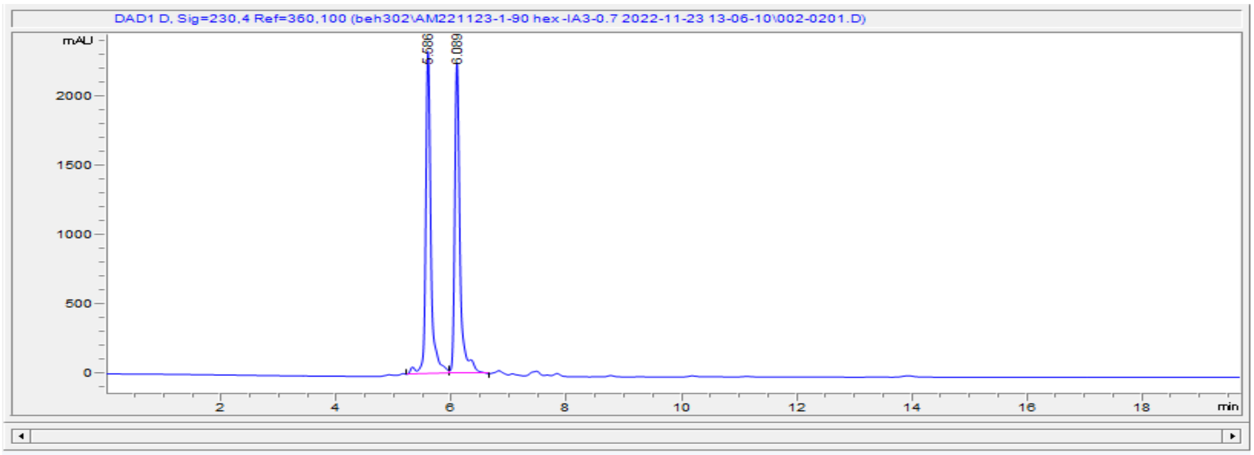

#	Time	Area	Height	Width	Area%	Symmetry
1	5.586	14837.1	2330.9	0.0951	50.750	0.781
2	6.089	14398.8	2244.5	0.0943	49.250	0.695

Figure S2. Chiral HPLC chromatogram of (S_P)- and (R_P)-**9** showing they form in a 1:1 ratio.

3. Photophysical Characterization

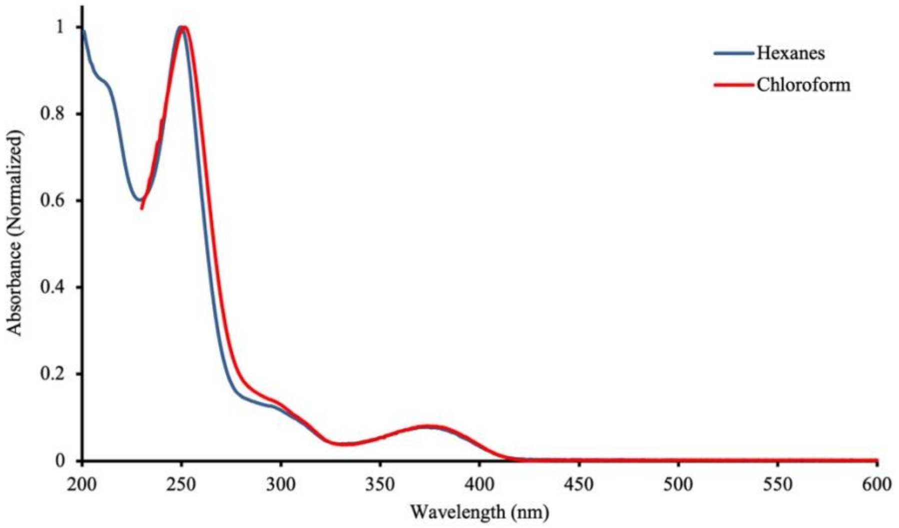

Figure S3. Absorbance of **9** in solution.

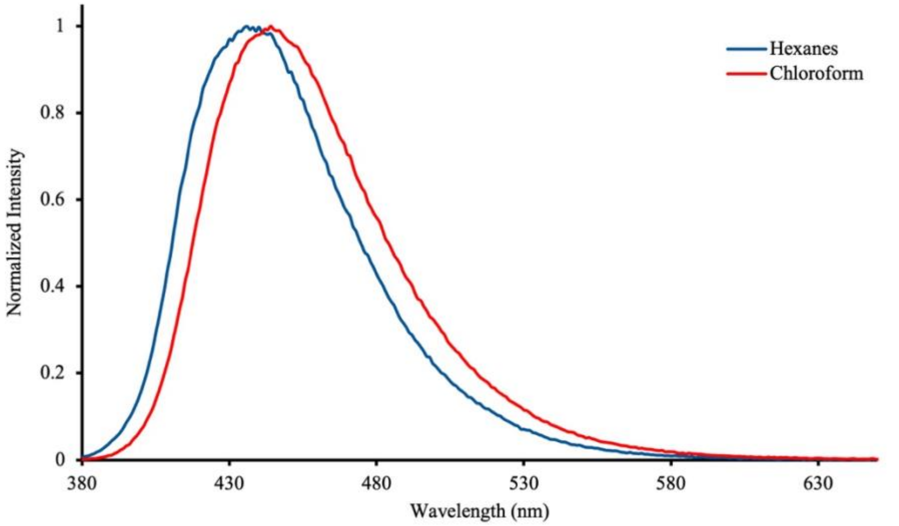

Figure S4. Emission of **9** in solution. (λ_{exc} = 373 nm for hexanes and λ_{exc} = 375 nm for chloroform)

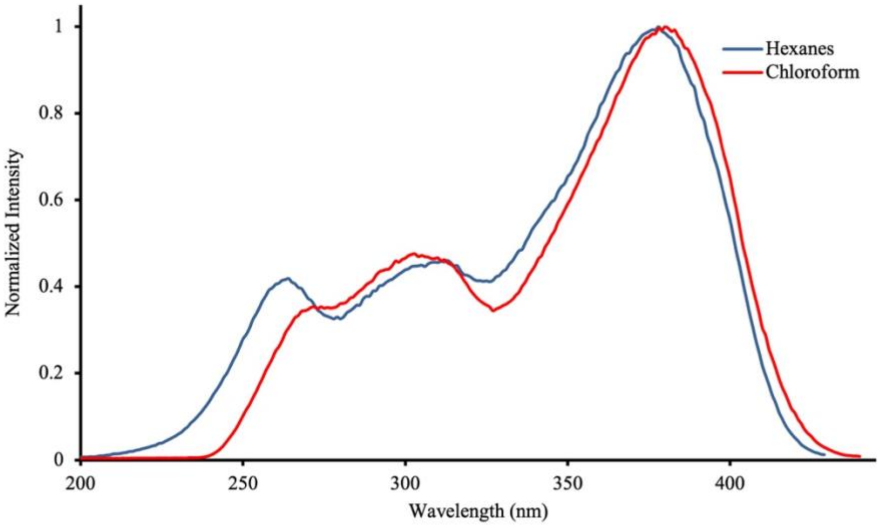

Figure S5. Excitation of **9** in solution. (λ_{em} = 438 nm for hexanes and λ_{em} = 444 nm for chloroform)

4. Screening Reactions With Olefin Metathesis Catalysts

General procedure for reaction screenings of **9** with olefin metathesis catalysts: In a nitrogen filled glovebox a stock solution of the desired initiator (10 mol%) was prepared in anhydrous, degassed THF or toluene. **9** (20 mg) was weighted out into a one-dram vial and brought into the glovebox, dissolved in anhydrous, degassed THF or toluene and transferred to an oven dried Schlenk tube. An appropriate amount of the catalyst solution was added for [**9**] = 100 mM. The Schlenk tube was sealed and removed from the glove box where it was subsequently wrapped in aluminum foil and kept at room temperature or placed in an oil bath at 50 ˚C or 100 ˚C and stirred. The reaction was cooled to room temperature and a large excess of deoxygenated ethyl vinyl ether (0.80 mL) was added and allowed to stir at room temperature for 12 hours. The reaction mixture was than concentrated down, allowed to dry under vacuum for one hour after which a ^{1}H NMR spectrum was recorded. Starting material was recovered by column chromatography.

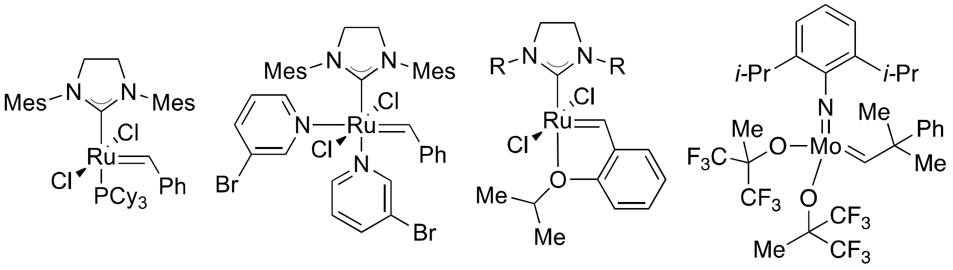

Scheme S1. Olefin Metathesis Catalysts screened.

Table S1. Reaction conditions screened for the reaction of **9** with olefin metathesis catalysts **11-15**.

Catalyst	[**9**]/[catalyst]	[**9**]	solvent	Temp (˚C)	Conv. after 24 hr
**12**	10	0.100	THF	50	NR
**12**	10	0.025	Toluene	100	NR
**11**	10	0.050	THF	50	NR
**11**	10	0.050	Toluene	100	NR
**13**	10	0.025	Toluene	100	NR
**14**	10	0.025	Toluene	100	NR
**15**	10	0.100	Toluene	rt	NR
**15**	10	0.100	Toluene	40	NR

5. *In situ* ^{1}H NMR experiments

Compound **9** as an inseparable mixture of enantiomers (30 mg, 0.041 mmol) and **12** or **15** were individually weighted out into 1 dram vials and brought into a nitrogen filled glovebox. **9** and the desired catalyst were separately dissolved in THF-d_8 or toluene-d_8 and combined for total volume of 0.418 mL ([**9**] = 100 mM) and transferred into a *J*-Young NMR tube that was sealed. The sample was removed from the glovebox, wrapped in aluminum foil, and placed in an ice bath. The sample was removed from the aluminum foil and placed into a 600 MHz NMR set to 25 ˚C from t = 0. For the sample with **12**, the spectrometer was then heated to 50 ˚C. ^{1}H NMR spectra were recorded every five minutes for the first hour and then every 20 minutes for 24 hours.

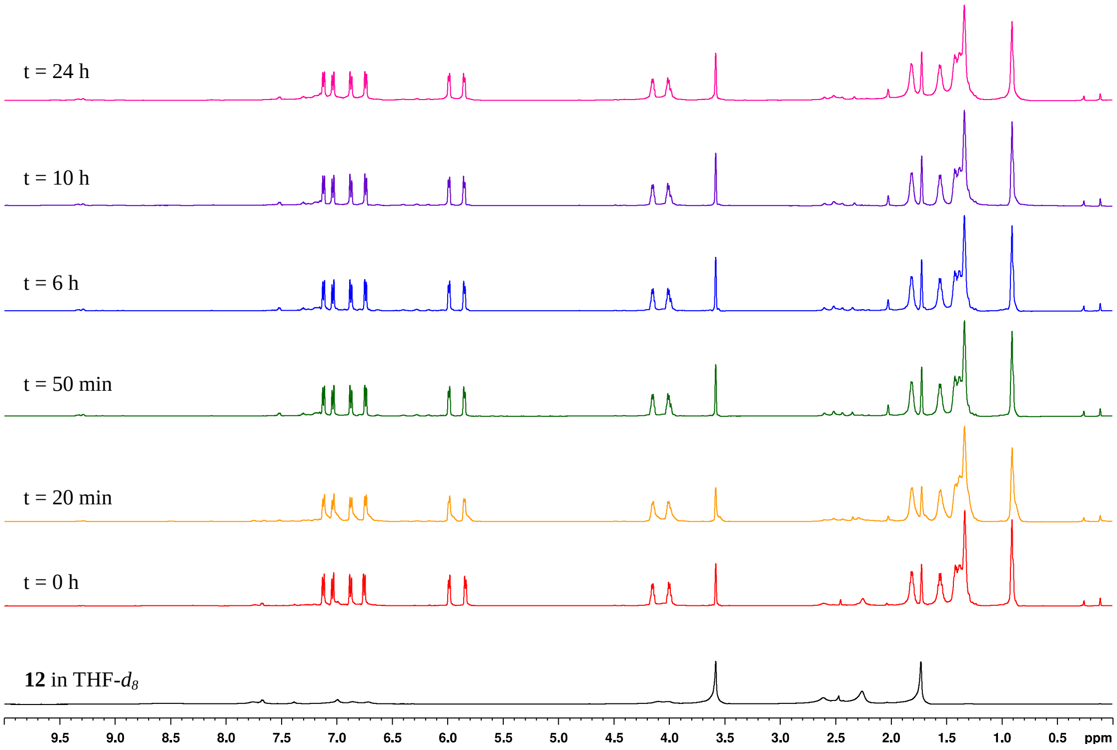

Figure S6. ^{1}H NMR reactivity experiment *in-situ* of **9** with Grubbs' third generation catalyst **12** at 50 ˚C in THF-d_8 showing no monomer consumption over time.

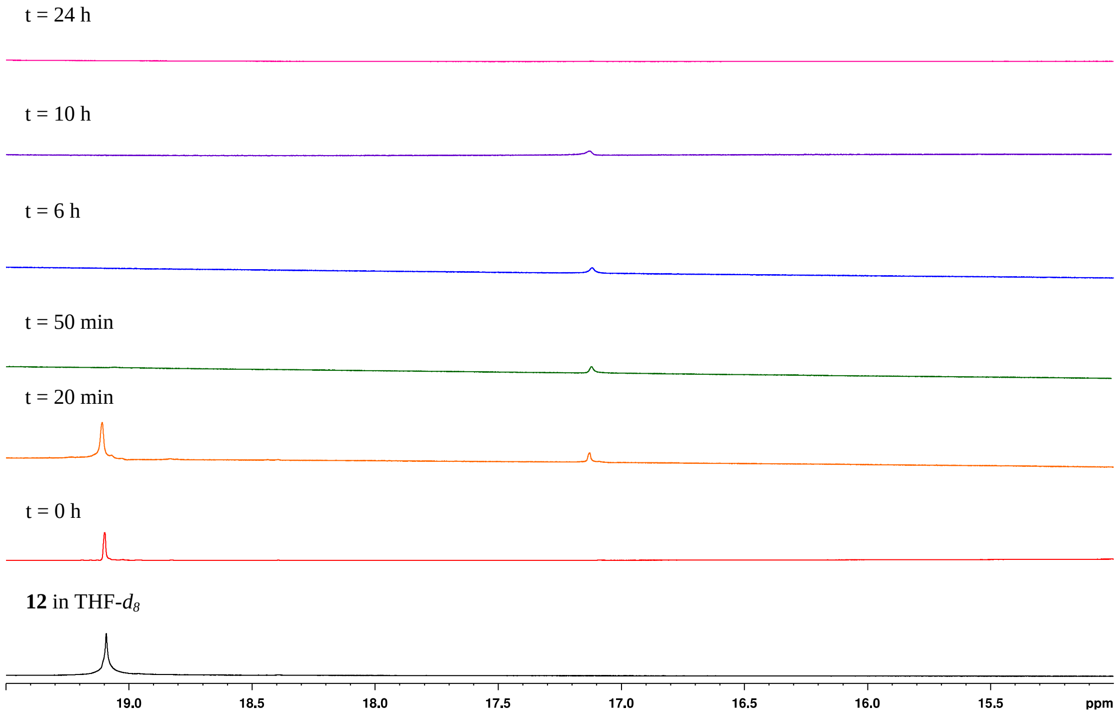

Figure S7. ^{1}H NMR reactivity experiment *in situ* of **9** with Grubbs' third generation catalyst **12** at 50 ˚C in THF-d_8 showing the shifts in the carbene region over time.

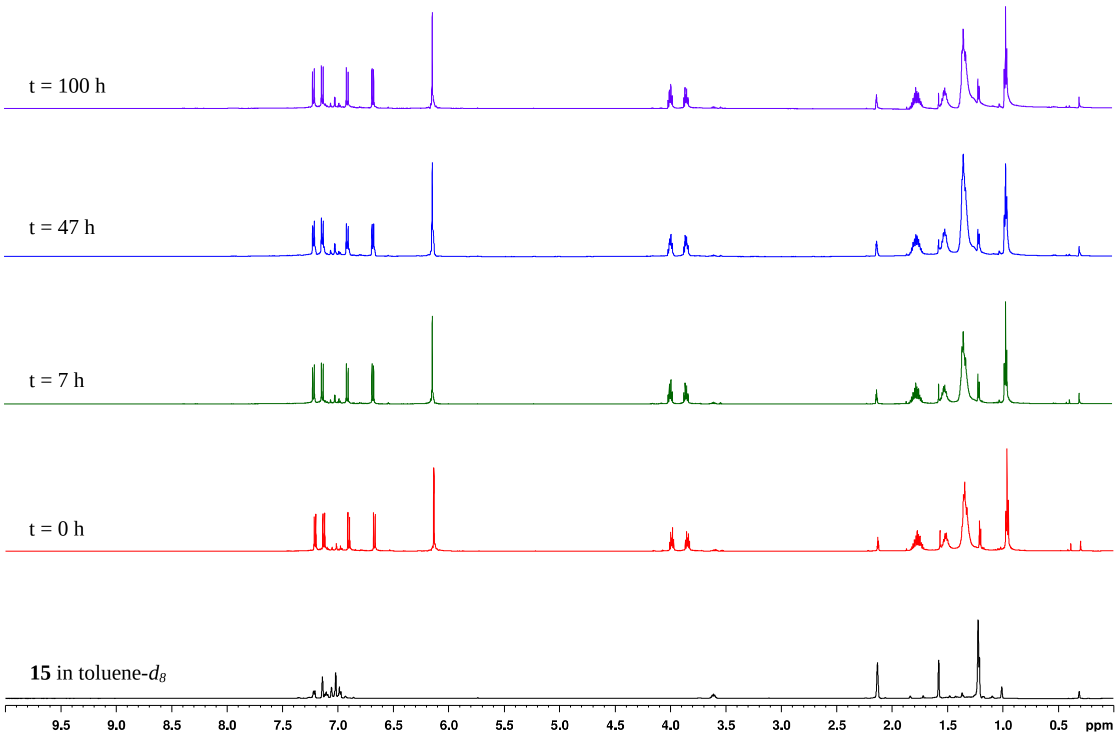

Figure S8. ^{1}H NMR reactivity experiment *in-situ* of **9** with Schrock's catalyst **15** at 50 ˚C in toluene-*d*8 showing no monomer consumption over time.

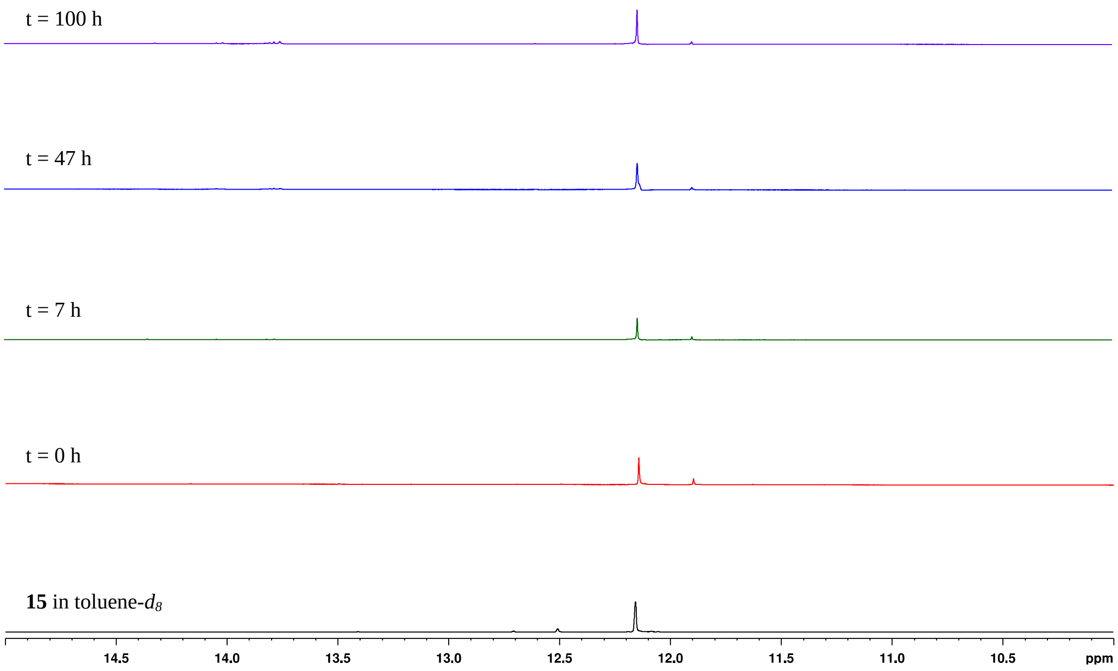

Figure S9. ^{1}H NMR reactivity experiment *in-situ* of **9** with Schrock's catalyst **15** at 25 ˚C in toluene-d_8 showing the shifts in the carbene region over time.

6. Strain Energy Calculations

7.1 Benchmarking

To accurately model strain energies ($\Delta G_{\text{ring strain}}$) of cyclophanes with density functional theory, benchmarking of basis sets and functionals were performed. Etheneolysis of **2** was modeled because the heat of etheneolysis, $\Delta G_{\text{etheneolysis}}$, is expected to correspond very closely to the ring strain such that:

$$\Delta G_{\text{ring strain}} = -\Delta G_{\text{etheneolysis}}$$

The ring strain for **2** has experimentally been determined to be 42.0 kcal/mol.[1]

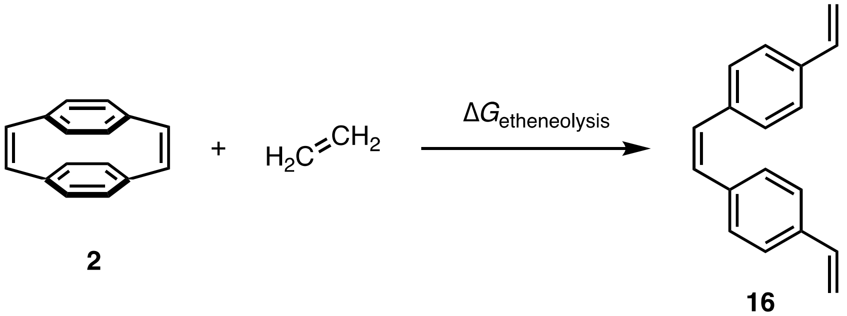

2

16

Scheme S2. Ethenolysis reaction used to model the strain energy of **2**

<u>Functional Screen</u>: Etheneolysis calculations were performed with Gaussian 16 (revision A.03)[2] at the DFT level of theory. Structures were constructed in Avogadro[3] and were pre-optimized using the UFF force field.[4] XYZ Coordinates of these structures were optimized in Gaussian using different functionals and the 6-31G* basis set[5][6] at a singlet spin state. The energies of these structures were calculated using single point calculations with the same functional and the 6-311G* basis set. These single point energies were used to determine the $\Delta G_{\text{etheneolysis}}$ via the following equation:

$$\Delta G_{\text{etheneolysis}} = E(\mathbf{16}) - (E(C_2H_4) + E(\mathbf{2}))$$

Where E is the single point energy of the optimized structure. The functionals that were screened were: M05, M06, M08HX, M11, MN15, B3LYP, O3LYP, X3LYP, B3P86, BH&HLYP, PW6B95, BMK, N12, TPSSh, ωB97X-D, LC-ωHPBE, APF, and APF-D. From these calculations, we obtained a spread of strain energies (Table S2, Figure S10).

Table S2. Ring strain energies and difference from accepted values for **2** at different functionals with the 6-311G* basis set.

Functional	$\Delta G_{\text{ring strain}}$ (kcal/mol)	Difference from accepted value (kcal/mol)
M11	44.5	2.5
M08HX	46.6	4.6
MN15	46.7	4.7
APFD	47.2	5.2
wB97XD	47.5	5.5
LC-wHPBE	48.0	6.0
M06	48.6	6.6
PW6B95	51.0	9.0
BMK	51.9	9.9
M05	52.1	10.1
APF	52.5	10.5
B3P86	52.6	10.6
TPSSh	52.6	10.6
N12	53.0	11.0
B3LYP	54.8	12.8
O3LYP	54.9	12.9
X3LYP	54.9	12.9
BH&HLYP	56.5	14.5

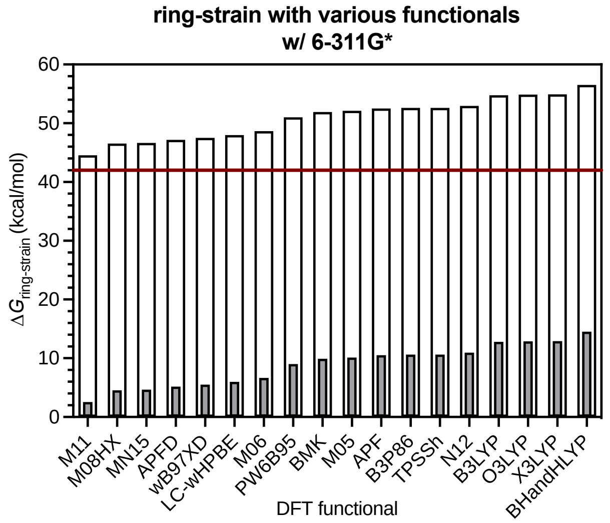

Figure S10. Ring strain energies (blue bars) and difference from accepted value (inset, grey bars) for **2** at different functionals – the experimental value of 42 kcal/mol is marked as a red horizontal line.

Based on these $\Delta G_{\text{ring strain}}$ values, the M11 functional was chosen as it only deviated from the accepted value by 2.5 kcal/mol. Following this, a basis set screen was performed with 6-311G, 6-311G*, aug-cc-pVDZ, cc-pVDZ, aug-cc-pVTZ, cc-pVTZ, def2-SVP, def2-TZV, def2-TZVP, def2-TZVPP and MidiX. From these calculations, we obtained a spread of strain energies (Table S3, Figure S11).

Table S3. Ring strain energies and difference from accepted values for **2** at different basis sets with the M11 functional.

Functional	$\Delta G_{\text{ring strain}}$ (kcal/mol)	Difference from accepted value (kcal/mol)
6-311G*	44.5	2.5
Aug-cc-pVDZ	45.8	3.8
cc-pVDZ	45.9	3.9
6-311G	46.1	4.1
Def2SVP	46.1	4.1
MidiX	46.5	4.5
Def2TZV	46.7	4.7
Def2TZVP	47.1	5.1
Def2TZVPP	47.4	5.4
Aug-cc-pVTZ	47.8	5.8
cc-pVTZ	47.9	5.9

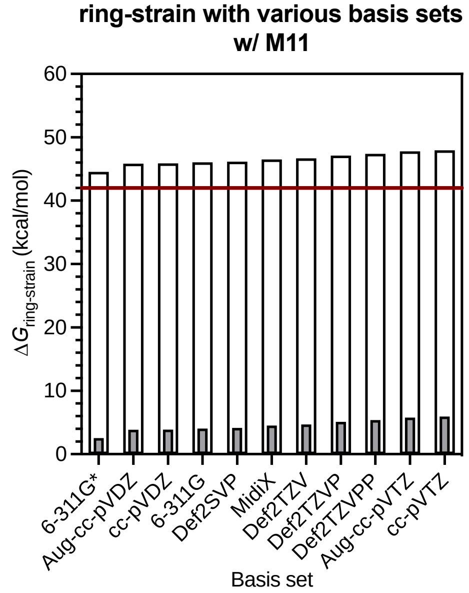

Figure S11. Ring strain energies (yellow bars) and difference from accepted value (inset, grey bars) for **2** at different functionals – the experimental value of 42 kcal/mol is marked as a red horizontal line.

Based on these Δ$G_{\text{ring strain}}$ values, M11/6-311G* level of theory is the level of theory that is most able to reproduce the experimentally determined 42.0 kcal/mol strain energy of **2**. As such, we proceeded with using M11/6-311G* as the level of theory for energy calculations.

7.2 Etheneolysis Calculations

Etheneolysis calculations were performed with Gaussian 16 (revision A.03)[2] at the DFT level of theory. Structures were constructed in Avogadro[3] and were pre-optimized using the UFF force field.[4] XYZ Coordinates of these structures were optimized in Gaussian using the M11 functional[10] and the 6-31G* basis set[5][6] at a singlet spin state. Optimized structures were confirmed from frequency calculations at the same level of theory, with all molecules described having no negative-valued vibrational modes. Energies of the optimized molecules were evaluated using single-point calculations at the M11/6-311G* level of theory. Finally, thermodynamic corrections were applied at 298 K using the entropy and enthalpy values obtained from the frequency calculations. The ring strain energy, Δ$G_{\text{ring strain}}$, was taken as the negative value of the heat of etheneolysis.

For compound **9**, etheneolysis of a truncated version (methoxy side-chains, not octyloxy) of the molecule, **17**, was used to determine the ring strain (Scheme S3).

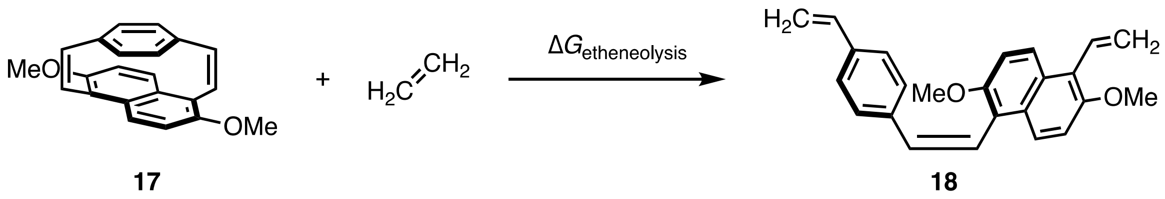

Scheme S3. Etheneolysis reaction used to determine the ring strain of **17**.

Δ$G_{\text{etheneolysis}}$(**17**) = –24.3 kcal/mol
Δ$G_{\text{ring strain}}$(**17**) = +24.3 kcal/mol

For comparison, the ring strain of a related compound, a dimethoxy paracyclophanediene (**19**) was computed via the same method (Scheme S4).

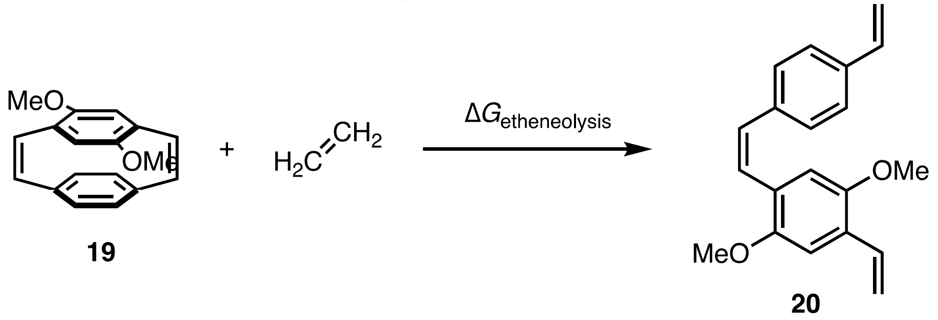

Scheme S4. Etheneolysis reaction used to determine the ring strain of **19**.

Δ$G_{\text{etheneolysis}}$(**19**) = –37.0 kcal/mol
Δ$G_{\text{ring strain}}$(**19**) = +37.0 kcal/mol

The strain energy of **17** is substantially lower than the strain energies of other cyclophanedienes such as **2**,[5] and **19** and is about the same as the strain energy of norbornene (24.7 kcal/mol).[6]

7.3 Strain Energy Decomposition Calculations

To evaluate the total strain in **17** and understand the unusually low $\Delta G_{\text{ring strain}}$ of **17**, we utilized an approach reported by Grimme[10] to decompose the cyclophane strain energy into meaningful components. Calculations were performed with Gaussian 16 (revision A.03)[2] at the DFT level of theory. Structures were constructed in Avogadro and pre-optimized in Avogadro using the UFF force field. XYZ Coordinates of these structures were optimized in Gaussian using the M11 functional and the 6-31G* basis set at a singlet spin state. Frequency calculations were not performed due to the presence of structures along the thermodynamic cycle used for energy decomposition analysis that do not correspond to equilibrium geometries (therefore, vibrational analysis would yield meaningless results). Energies of the optimized molecules were evaluated using single-point calculations at the M11/6-311G* level of theory.

The energy decomposition analysis described breaks the total strain energy, $\Delta E_{\text{total strain}}$, into a sum of four components that arise from isodesmic reactions along a complete etheneolysis reaction path:

$$-\Delta E_{\text{total strain}} = \Delta E_{\text{total etheneolysis}} = \Delta E_{\text{dealkenylation}} + \Delta E_{\text{alkenylation}} + \Delta E_{\pi\text{-interaction}} + \Delta E_{\text{arene strain}}$$

Where $\Delta E_{\text{dealkenylation}}$ corresponds to the energy of replacing the bridging alkenes with H atoms (but keeping the arenes in the same geometry), $\Delta E_{\pi\text{-interaction}}$ corresponds to the energy of separating the two bent arenes, $\Delta E_{\text{arene strain}}$ corresponds to the energy of relaxing the arenes from their bent geometries to their flat, equilibrium geometries, and $\Delta E_{\text{alkenylation}}$ corresponds to the energy penalty of replacing the alkenes (yielding complete etheneolysis products). The reactions used to calculate these energies for **17** are depicted in Scheme S5.

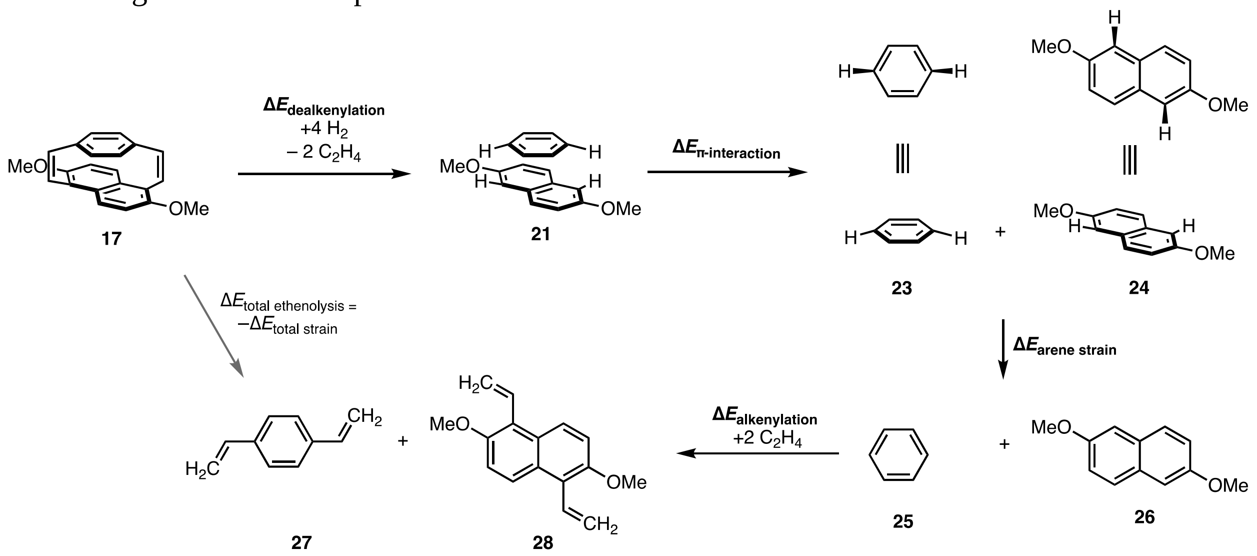

Scheme S5. Thermodynamic cycle corresponding to energy decomposition of $\Delta E_{\text{total strain}}$ of **17**.

To fully understand the values of $\Delta E_{\text{dealkenylation}}$, $\Delta E_{\text{alkenylation}}$, $\Delta E_{\pi\text{-interaction}}$, and $\Delta E_{\text{arene strain}}$ of **17** in the context of other cyclophanes, we also calculated these values for compound **19** via a similar cycle (Scheme S6). The values obtained from both thermodynamic cycles are in Table S4.

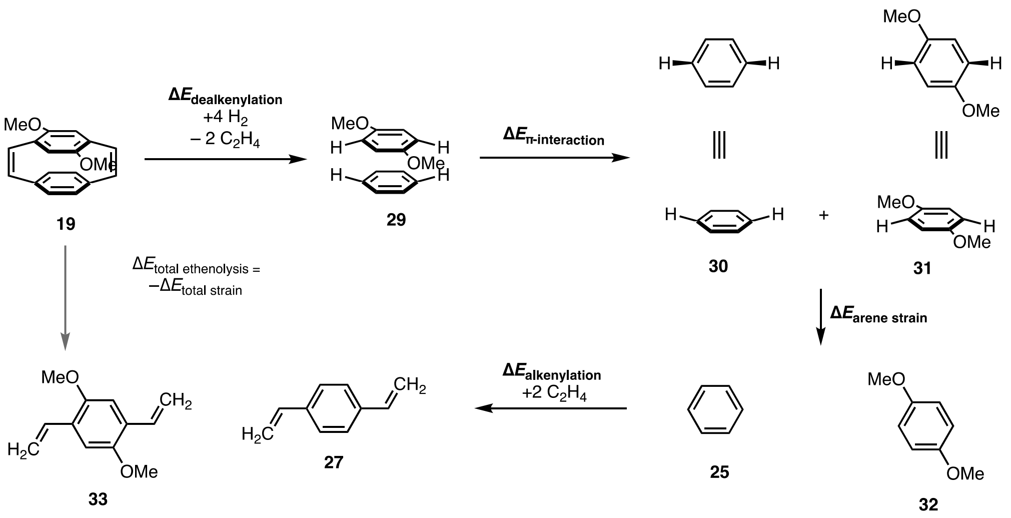

Scheme S6. Thermodynamic cycle corresponding to energy decomposition of $\Delta E_{\text{total strain}}$ of **17**. *Note: although **30** is visually similar to **23**, the 3-dimensional geometries of **30** and **23** are different because they arise from different cyclophanes.

Table S4: Decomposition of the $\Delta E_{\text{total strain}}$ for **17** and **19**. Energies are in kcal/mol.

	Energies for **17** (kcal/mol)	Energies for **19** (kcal/mol)	Difference ΔE(**19**) – ΔE(**17**)
$\Delta E_{\text{dealkenylation}}$	– 20.5	– 38.5	– 17.9
$\Delta E_{\pi\text{-interaction}}$	– 7.4	– 6.0	+ 1.4
$\Delta E_{\text{arene strain}}$	– 40.6	– 33.6	+ 7.0
$\Delta E_{\text{alkenylation}}$	+ 40.3	+ 34.9	– 5.4
$\boldsymbol{\Delta E_{\text{total etheneolysis}}}$	– 28.2	– 43.1	– 14.9
$\boldsymbol{\Delta E_{\text{total strain}}}$	**+ 28.2**	**+ 43.1**	**+ 14.9**

The $\Delta E_{\text{total strain}}$ is 14.9 kcal/mol smaller in **17** than in **19**. While this result was surprising given the size mismatch of the arenes in **17** and the highly deformed naphthalene, the energy decomposition helps to rationalize this result. First, the $\Delta E_{\text{dealkyenylation}}$ is 18.0 kcal/mol smaller in **17** than in **19**, meaning the transformation from **17** to **21** is less favorable than the transformation of **19** to **29** (Scheme S7).

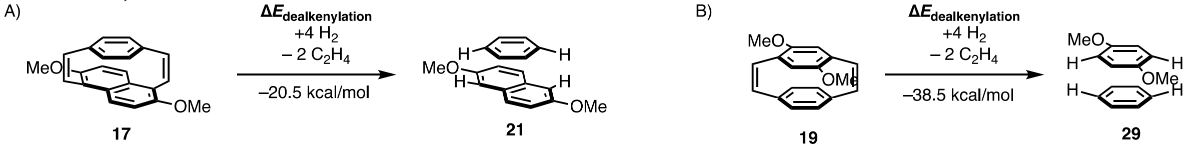

Scheme S7. The dealkenylation steps for energy decomposition analysis of A) **17** and B) **19**.

This difference in $\Delta E_{\text{dealkyenylation}}$ is rationalized from a structural perspective: in **19**, the bridging alkenes make a 90º dihedral angle with the place of the benzenes, preventing any π-conjugation from occurring. In **17**, the alkene bridges make a 65º dihedral angle (Figure S12), enabling some amount of *stabilizing* π-conjugation between the alkene bridges and arenes, making $\Delta E_{\text{dealkyenylation}}$ less favorable in **17** and reducing the total strain.

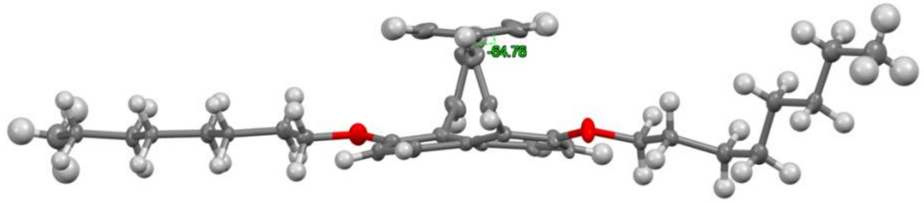

Figure S12. Single crystal XRD structure of **9,** oriented to highlight the ~65º dihedral angle between the arenes and alkene bridges.

Next, the $\Delta E_{\pi\text{-interaction}}$ is more negative for **17** than **19**, indicating that separating the two arenes in structure **21** is more favorable than in structure **29** (Scheme S8).

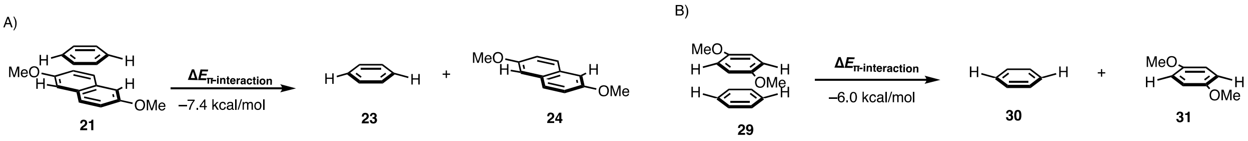

Scheme S8. The π-interaction evaluation step for energy decomposition analysis of A) **17** and B) **19**.

Given that structures in **17** and **19** are donor/acceptor systems, the more favorable $\Delta E_{\pi\text{-interaction}}$ in **17** is rationalized on the basis of the arenes in **21** are slightly closer ($d_{\text{centroid-to-centroid}}$(**17**) = 2.948 Å) than the arenes in **19** ($d_{\text{centroid-to-centroid}}$(**19**) = 2.981 Å) causing slightly more unfavorable π-interactions in **21** than in **29** and overall adding *slightly* to the ring strain of **17** compared to **19**.

Next, the $\Delta E_{\text{arene strain}}$ is 7.0 kcal/mol more negative for **17** than **19**, indicating that in sum the arenes are more strained in **17** (Scheme S8). This is likely due to the large size mismatch between the benzene and the naphthalene in **17**, evidenced by the H-to-H distances (d_{HH}) in the equilibrium geometries of **25** (d_{HH} = 4.96 Å) and **26** (d_{HH} = 5.59 Å) with a Δd_{HH} = 0.63 Å, while the sizes of the two arenes in **19** are well-matched, with a Δd_{HH} = 0.01 Å because both are benzene. This mismatch in size contributes to the ring strain of **17** compared to **19**.

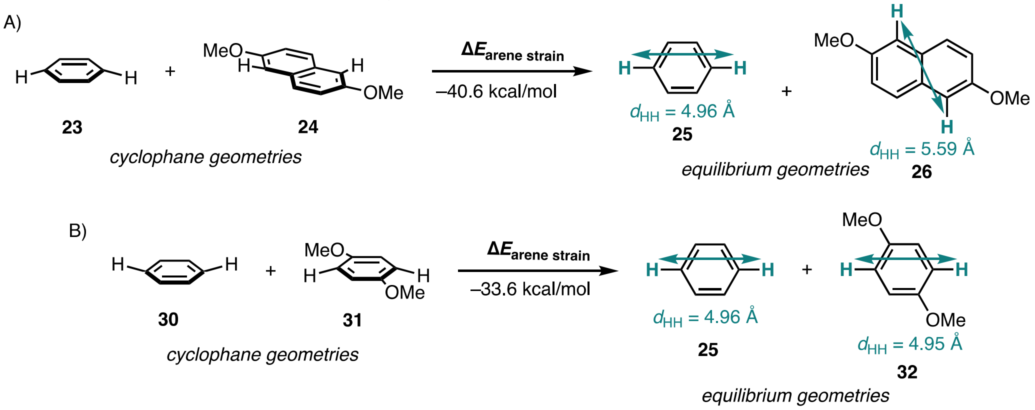

Scheme S9. The arene-strain evaluation step for energy decomposition

analysis of A) **17** and B) **19**.

Lastly, the $\Delta E_{\text{alkenylation}}$ is 4.46 kcal/mol more positive in **17** than in **19**, indicating that alkenylation is less favorable in **17** than **19**.

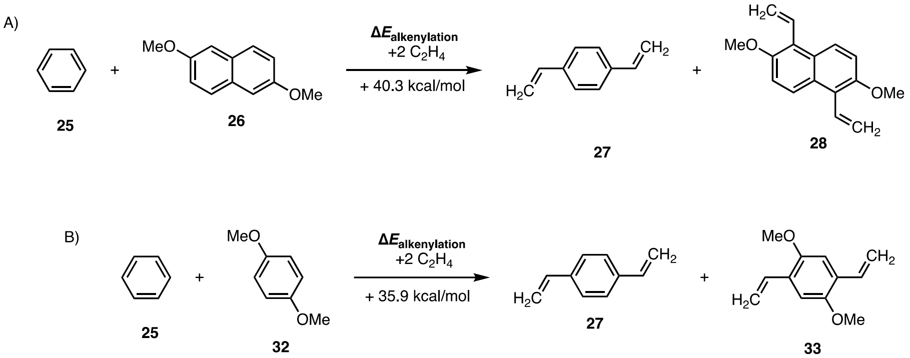

Scheme S10. The alkenylation step for energy decomposition analysis of A) **17** and B) **19**.

We ascribe this to the unfavorable steric interactions present in the product **28** due to the substitution pattern of the naphthalene. The torsional angle between the arene and alkenes in **28** is approximately 38º (Figure S13). In **27** and **33**, the same torsional angle between the arene and alkene is nearly 0º. This deviation from planarity in **28** makes the alkenylation step in **17** less favorable because it reduces the conjugation between the arene an alkene. As such, the $\Delta E_{\text{alkenylation}}$ for **17** is more positive than in **19**, reducing the driving force for ring-opening in **17** and therefore reducing the strain energy in **17**.

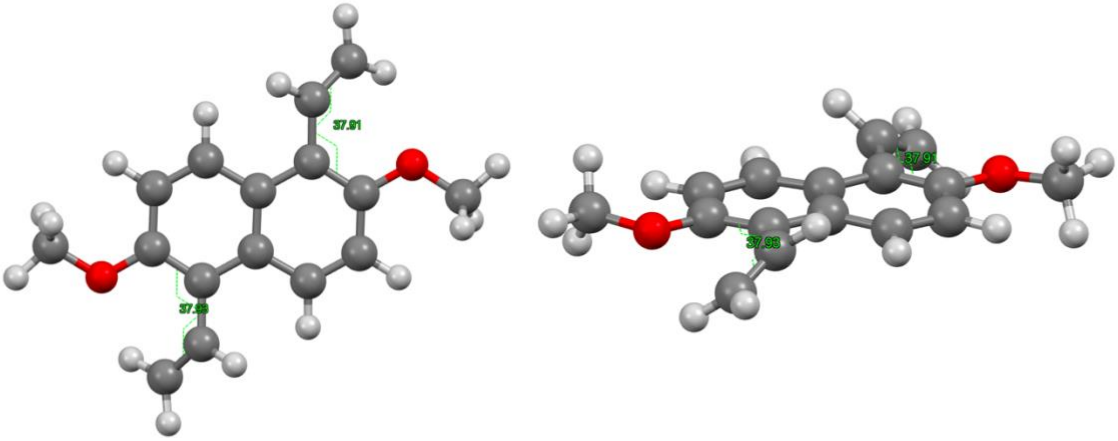

Figure S13. Top-down and side view of **28**, highlighting the non-planar geometry caused by substituent steric repulsion.

Combined, these results demonstrate that the largest contributor to the reduced strain of **17** compared to **19** is the twisting of the rings that enables conjugation between the olefins and arenes, stabilizing **17**. This is evidenced by the less negative $\Delta E_{\text{dealkenylation}}$ in **17** compared to **19**. The effect of mismatched arene sized was minimal from a strain-energy perspective, given that the difference in arene strain caused by the mismatched size (7.0 kcal/mol *additional* strain) was nearly as large as the effect of steric repulsion between substituents in the product (5.4 kcal/mol *less* strain). In arenes with different substitution patterns (in which the products do not have torsional strain, like

the strain present in **28**) we expect the strain energy can be increased relative to **17**. It is unlikely, however, that other cyclophanedienes with size mis-matched arenes will have larger strain energy than cyclophanedienes in which both arenes are benzene, because the size-mismatch between the arenes will contribute to twisting of the rings which reduces the dihedral angle between the olefins and arenes, reducing the strain energy.

7. Calculations with Ruthenium Catalysts

In the main text, we noted that **9** is unable to undergo ring-opening metathesis polymerization with olefin metathesis catalysts. We rationalized that this is because the hydrogens at the C4 and C7 positions block the olefin metathesis catalyst from coordinating to the olefins of **9**. To understand the steric environment of such olefin metathesis catalysts while π-bound to the cyclophanes, we simulated Grubbs' second-generation initiator coordinated to the olefins of **17**. We also performed the same analysis with the catalyst bound to **19**, because monomers with similar structures to **19** are easily polymerized via ROMP.

Calculations were performed with Gaussian 16 (revision A.03) at the DFT level of theory. Structures were constructed in Avogadro and pre-optimized in Avogadro using the UFF force field. XYZ Coordinates of these structures were optimized in Gaussian using the M11 functional and the 6-31G* basis set at a singlet spin state. The geometry and relevant distances are shown in Figures S14 and S15.

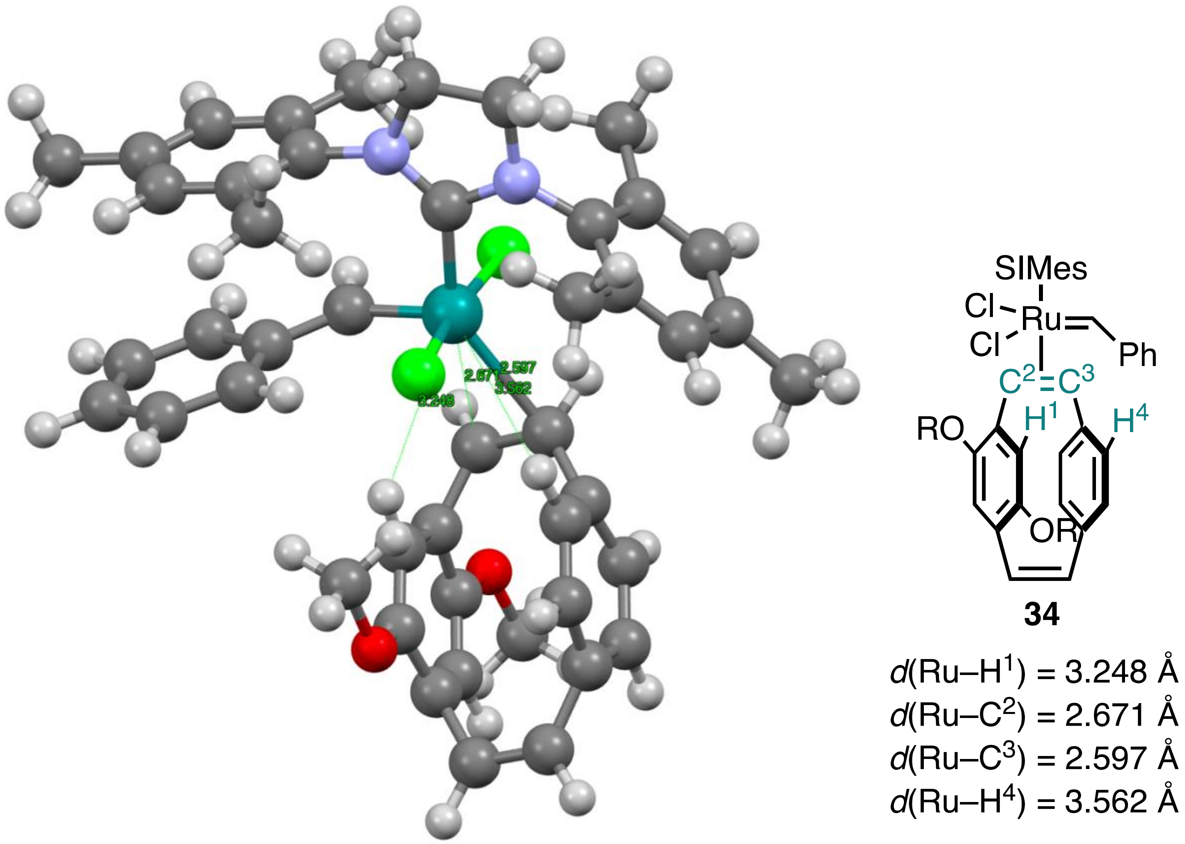

Figure S14. Geometry and relevant distances to Ru of **34**, the complex between Grubbs' second-generation initiator and cyclophane **19**.

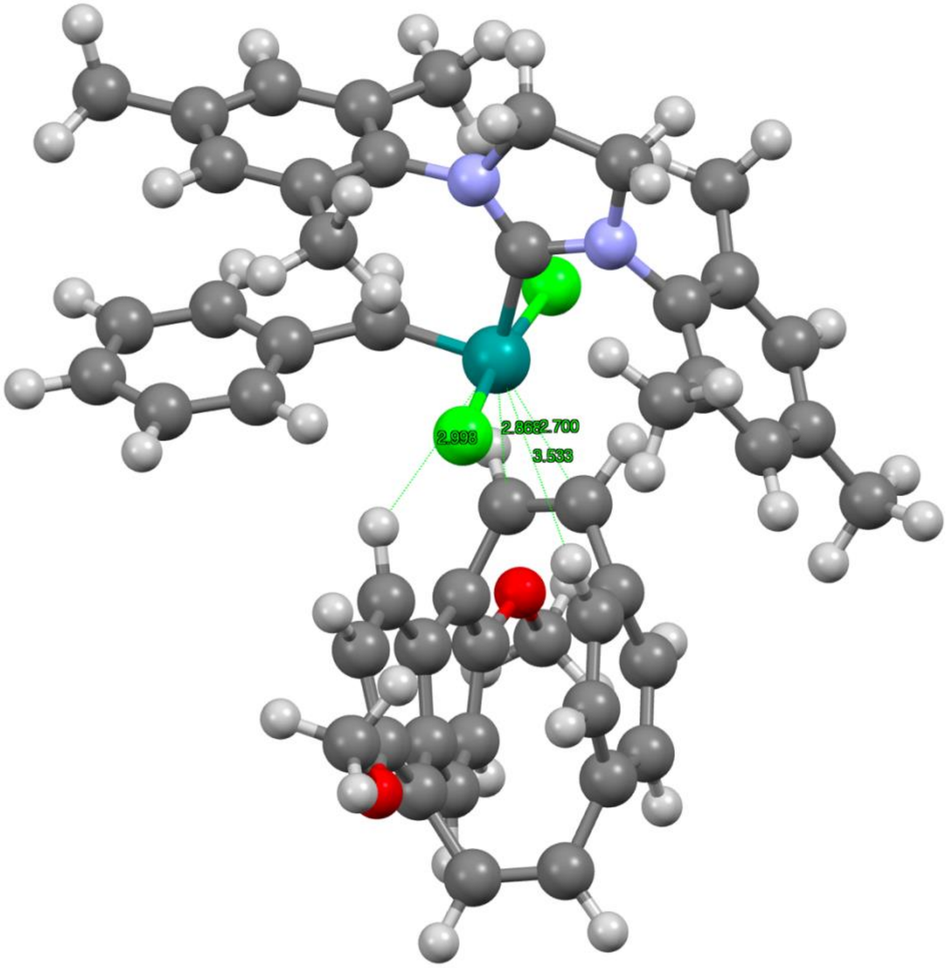

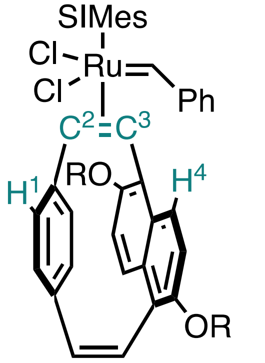

35

d(Ru–H^1) = 3.533 Å
d(Ru–C^2) = 2.700 Å
d(Ru–C^3) = 2.862 Å
d(Ru–H^4) = 2.998 Å

Figure S15. Geometry and relevant distances to Ru of **35**, the complex between Grubbs' second-generation initiator and cyclophane **17**.

In complex **34**, the Ru–C distances are d(Ru–C^2) = 2.671 Å and d(Ru–C^3) = 2.597 Å, while in complex **35**, the Ru–C distances are longer at d(Ru–C^2) = 2.700 Å and d(Ru–C^2) = 2.862 Å. The longer Ru–C distances in **35** compared to **34** are accompanied by shorter Ru–H distances to the closest H atoms, with the shortest Ru–H distance in **34** being d(Ru–H^1) = 3.248 Å, and the shortest Ru–H distance in **35** is d(Ru–H^4) = 2.998 Å. Taken together, we believe that the shorter Ru–H distance in **35** arises from steric blocking of the Ru by the H atoms on the naphthalene moiety in **9**, leading to worse binding of Ru to the olefins and consequentially, longer Ru–C distances in **35** compared to **34**. The weaker binding of the Ru catalyst to **9** likely prevents the formation of the metallacyclobutadiene needed to facilitate olefin metathesis (and subsequent polymerization). This hypothesis is consistent with the spectra discussed in SI section 6, Figure S7, in which new carbene resonance appear in ^{1}H NMR spectra after the addition of monomer to Grubbs catalysts, indicating binding of the Ru to the olefins of **9**, but the new resonances quickly decrease in intensity as the Ru catalysts decomposes due to the impeded metallacyclobutane formation.

8. X-Ray Crystallographic Data

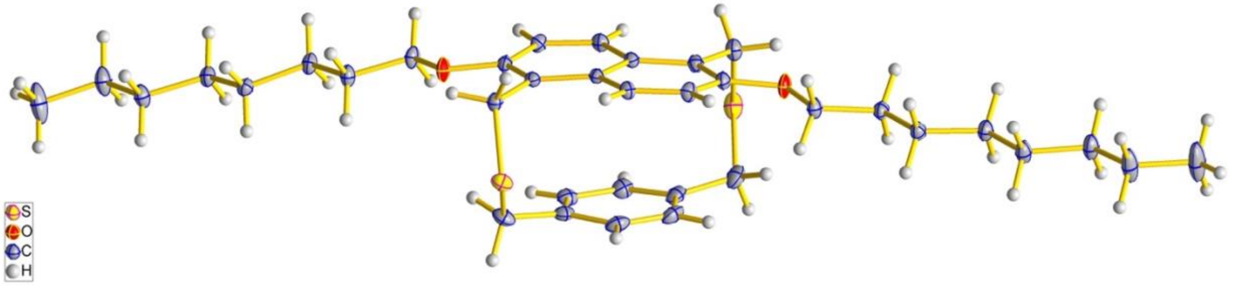

Figure S16. The molecular structure of dithia[3.3]naphthalenoparacyclophane **6** (ellipsoids set at 50% probability).

A colorless, block-like specimen of $C_{36}H_{50}O_2S_2$, approximate dimensions 0.360 mm x 0.500 mm x 0.540 mm, was used for the X-ray crystallographic analysis. The X-ray intensity data were measured on a Bruker D8 SMART APEXII three-circle diffractometer system equipped with a Incotec microfocus sealed X-ray tube (MoKα, λ = 0.71073 Å) and a multilayer optics monochromator.

A total of 1620 frames were collected. The total exposure time was 2.06 hours. The frames were integrated with the Bruker SAINT software package using a narrow-frame algorithm. The integration of the data using a monoclinic unit cell yielded a total of 57951 reflections to a maximum θ angle of 28.34° (0.75Å resolution), of which 8030 were independent (average redundancy 7.217, completeness = 99.8%, R_{int} = 4.95%, R_{sig} = 3.17%) and 6656 (82.89%) were greater than $2\sigma(F^2)$. The final cell constants of a = 14.4587(4) Å, b = 18.9731(5) Å, c = 11.8003(3) Å, β = 94.4444(13)°, volume = 3227.40(15) $Å^3$, are based upon the refinement of the XYZ-centroids of 9872 reflections above 20 σ(I) with 4.802° < 2θ < 56.42°. Data were corrected for absorption effects using the Multi-Scan method (SADABS). The ratio of minimum to maximum apparent transmission was 0.956. The calculated minimum and maximum transmission coefficients (based on crystal size) are 0.9020 and 0.9330.

The structure was solved and refined using the Bruker SHELXTL Software Package, using the space group P 1 21/c 1, with Z = 4 for the formula unit, $C_{36}H_{50}O_2S_2$. The final anisotropic full-matrix least-squares refinement on F^2 with 363 variables converged at R1 = 3.80%, for the observed data and wR2 = 10.17% for all data. The goodness-of-fit was 1.084. The largest peak in the final difference electron density synthesis was 0.341 $e^-/Å^3$ and the largest hole was -0.340 $e^-/Å^3$ with an RMS deviation of 0.049 $e^-/Å^3$. On the basis of the final model, the calculated density was 1.191 g/cm^3 and F(000), 1256e^-.

Table S5. Sample and crystal data for dithia[3.3]naphthalenoparacyclophane **6**.

**Identification code**	23maw1h	
**Chemical formula**	$C_{36}H_{50}O_2S_2$	
**Formula weight**	578.88 g/mol	
**Temperature**	100(2) K	
**Wavelength**	0.71073 Å	
**Crystal size**	0.360 x 0.500 x 0.540 mm	
**Crystal habit**	colorless block	
**Crystal system**	monoclinic	
**Space group**	P 1 21/c 1	
**Unit cell dimensions**	a = 14.4587(4) Å	α = 90°
	b = 18.9731(5) Å	β = 94.4444(13)°
	c = 11.8003(3) Å	γ = 90°
**Volume**	3227.40(15) $Å^3$	
**Z**	4	
**Density (calculated)**	1.191 g/cm^3	
**Absorption coefficient**	0.195 mm^{-1}	
**F(000)**	1256	

Table S6. Data collection and structure refinement details for dithia[3.3]naphthaleno-paracyclophane **6**

**Diffractometer**	Bruker D8 SMART APEXII three-circle diffractometer
**Radiation source**	Incotec microfocus sealed X-ray tube (MoKα, λ = 0.71073 Å)
**Theta range for data collection**	1.77 to 28.34°
**Index ranges**	-19<=h<=17, -25<=k<=25, -15<=l<=15
**Reflections collected**	57951
**Independent reflections**	8030 [R(int) = 0.0495]
**Coverage of independent reflections**	99.8%
**Absorption correction**	Multi-Scan
**Max. and min. transmission**	0.9330 and 0.9020
**Structure solution technique**	direct methods
**Structure solution program**	SHELXT (Sheldrick 2015)
**Refinement method**	Full-matrix least-squares on F^2
**Refinement program**	SHELXL-2018/3 (Sheldrick, 2018)
**Function minimized**	Σ w(F_o^2 - F_c^2)^2
**Data / restraints / parameters**	8030 / 0 / 363
**Goodness-of-fit on F^2**	1.084
**Δ/σ_{max}**	0.001
**Final R indices**	6656 data; I>2σ(I) R1 = 0.0380, wR2 = 0.0977
	all data R1 = 0.0467, wR2 = 0.1017
**Weighting scheme**	w=1/[σ^2(F_o^2)+(0.0488P)^2+0.7797P]<br>where P=(F_o^2+2F_c^2)/3
**Largest diff. peak and hole**	0.341 and -0.340 eÅ^{-3}
**R.M.S. deviation from mean**	0.049 eÅ^{-3}

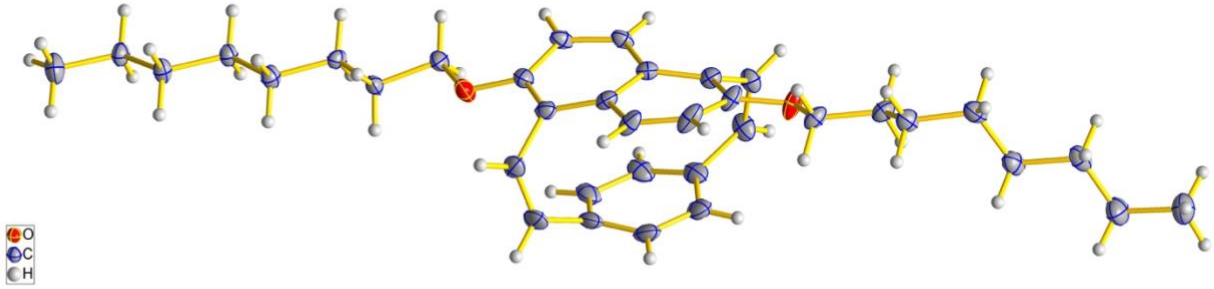

Figure S17. The molecular structure of [2.2]naphthalenoparacyclophanediene **9** (ellipsoids set at 50% probability).

A colorless, block-like specimen of $C_{36}H_{46}O_2$, approximate dimensions 0.300 mm x 0.370 mm x 0.560 mm, was used for the X-ray crystallographic analysis. The X-ray intensity data were measured on a Bruker D8 SMART APEXII three-circle diffractometer system equipped with a Incotec microfocus sealed X-ray tube (MoKα, λ = 0.71073 Å) and a multilayer optics monochromator.

A total of 2609 frames were collected. The total exposure time was 5.46 hours. The frames were integrated with the Bruker SAINT software package using a narrow-frame algorithm. The integration of the data using a triclinic unit cell yielded a total of 41732 reflections to a maximum θ angle of 28.32° (0.75 Å resolution), of which 7257 were independent (average redundancy 5.751, completeness = 99.8%, R_{int} = 3.73%, R_{sig} = 2.91%) and 5916 (81.52%) were greater than $2\sigma(F^2)$. The final cell constants of a = 10.1306(5) Å, b = 12.1662(6) Å, c = 12.8685(6) Å, α = 98.5268(16)°, β = 94.4721(16)°, γ = 109.9084(15)°, volume = 1460.64(12) $Å^3$, are based upon the refinement of the XYZ-centroids of 9874 reflections above 20 σ(I) with 4.539° < 2θ < 56.58°. Data were corrected for absorption effects using the Multi-Scan method (SADABS). The ratio of minimum to maximum apparent transmission was 0.970. The calculated minimum and maximum transmission coefficients (based on crystal size) are 0.9620 and 0.9790.

The structure was solved and refined using the Bruker SHELXTL Software Package, using the space group P -1, with Z = 2 for the formula unit, $C_{36}H_{46}O_2$. The final anisotropic full-matrix least-squares refinement on F^2 with 410 variables converged at R1 = 4.64%, for the observed data and wR2 = 12.51% for all data. The goodness-of-fit was 1.064. The largest peak in the final difference electron density synthesis was 0.315 $e^-/Å^3$ and the largest hole was -0.205 $e^-/Å^3$ with an RMS deviation of 0.039 $e^-/Å^3$. On the basis of the final model, the calculated density was 1.161 g/cm^3 and F(000), 556 e^-.

Table S7. Sample and crystal data for [2.2]naphthalenoparacyclophanediene 9.

**Identification code**	23maw2h	
**Chemical formula**	$C_{36}H_{46}O_2$	
**Formula weight**	510.73 g/mol	
**Temperature**	100(2) K	
**Wavelength**	0.71073 Å	
**Crystal size**	0.300 x 0.370 x 0.560 mm	
**Crystal habit**	colorless block	
**Crystal system**	triclinic	
**Space group**	P -1	
**Unit cell dimensions**	a = 10.1306(5) Å	α = 98.5268(16)°
	b = 12.1662(6) Å	β = 94.4721(16)°
	c = 12.8685(6) Å	γ = 109.9084(15)°
**Volume**	1460.64(12) Å^3	
**Z**	2	
**Density (calculated)**	1.161 g/cm^3	
**Absorption coefficient**	0.070 mm^{-1}	
**F(000)**	556	

Table S8. Data collection and structure refinement details for [2.2]naphthalenoparacyclophanediene **9**.

**Diffractometer**	Bruker D8 SMART APEXII three-circle diffractometer
**Radiation source**	Incotec microfocus sealed X-ray tube (MoKα, λ = 0.71073 Å)
**Theta range for data collection**	2.16 to 28.32°
**Index ranges**	-13<=h<=13, -16<=k<=16, -17<=l<=16
**Reflections collected**	41732
**Independent reflections**	7257 [R(int) = 0.0373]
**Coverage of independent reflections**	99.8%
**Absorption correction**	Multi-Scan
**Max. and min. transmission**	0.9790 and 0.9620
**Structure solution technique**	direct methods
**Structure solution program**	SHELXT (Sheldrick 2015)
**Refinement method**	Full-matrix least-squares on F^2
**Refinement program**	SHELXL-2018/3 (Sheldrick, 2018)
**Function minimized**	$\Sigma w(F_o^2 - F_c^2)^2$
**Data / restraints / parameters**	7257 / 24 / 410
**Goodness-of-fit on F^2**	1.064
**Δ/σ_{max}**	0.001
**Final R indices**	5916 data; I>2σ(I) R1 = 0.0464, wR2 = 0.1196
	all data R1 = 0.0558, wR2 = 0.1251
**Weighting scheme**	$w=1/[\sigma^2(F_o^2)+(0.0564P)^2+0.3944P]$<br>where $P=(F_o^2+2F_c^2)/3$
**Largest diff. peak and hole**	0.315 and -0.205 eÅ^{-3}
**R.M.S. deviation from mean**	0.39 $^{-3}$

9. Determining the angle of α and β

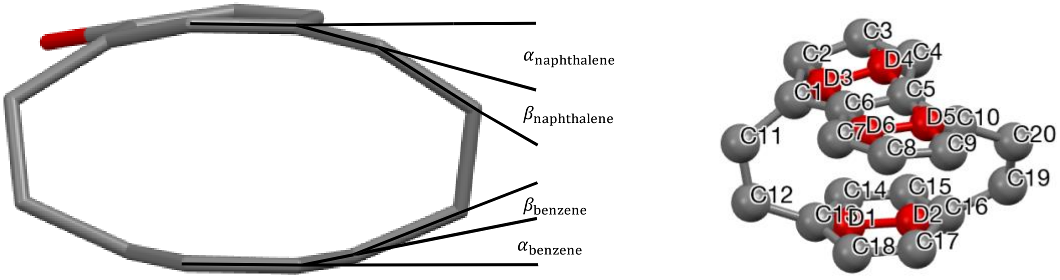

Figure S18. Representation of angles α and β (left) and insertion of dummy atoms to the crystal structure of **9** to calculate the angles of α and β (right).

REM dummy position of the center between C14 and C18
D1 3 0.5405325 0.552812 0.249038 11.00000 0.02312
REM C12-C13-D1 167.4 degrees, beta=180-167.4=12.6
REM C13-D1-D2 169.5 degrees, alpha=180-169.5=10.5

D2 3 0.5140045 0.432109 0.2463155 11.00000 0.02603
REM C19-C16-D2 166.4 degrees, beta=180-166.4=13.6
REM C16-D2-D1 169.4 degrees, alpha=180-169.4=10.6

REM dummy position of the center between C2 and C6
D3 3 0.8517285 0.6199305 0.1957515 11.00000 0.03218
REM dummy position of the center between C3 and C5
D4 3 0.8365345 0.4999185 0.161083 11.00000 0.04431
REM C11-C1-D3 164.5 degrees, beta=180-164.5=15.5
REM C1-D3-D4 168.8 degrees, alpha=180-168.8=11.2

REM dummy position of the center between C5 and C9
D5 3 0.8211635 0.4641415 0.3403625 11.00000 0.01974
REM dummy position of the center between C6 and C8
D6 3 0.861047 0.587954 0.3769935 11.00000 0.01653
REM C20-C10-D5 164.6 degrees, beta=180-164.6=15.4
REM C10-D5-D6 169.5 degrees, alpha=180-169.5=10.5

10. XYZ coordinates of Computed Structures

The format of the XYZ structures below is as follows:

Chemdraw image

First line = the number of atoms in the structure

Second line = a comment line which contains the compound number and the computed electronic energy in hartree and any thermodynamic corrections

Third line onward = the element, x-coordinate, y-coordinate, and z-coordinate, in Å.

This document is constructed so that structures can be opened in molecular editors that can read .xyz files, such as MOLDEN or Avogadro.

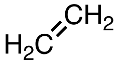

6

Ethylene, E = -78.54154444 Ha, ΔH = 34.008 kcal/mol, ΔS = 53.705 cal/mol•K

H	2.955497	-0.010223	-3.099285
C	3.232054	0.776869	-3.803381
C	4.322237	1.514653	-3.631178
H	2.567230	0.926882	-4.656178
H	4.987057	1.364641	-2.778383
H	4.598795	2.301748	-4.335275

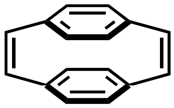

28

2, E = -616.430446409 Ha

C	-0.696511	1.195658	1.554115
C	-1.401868	-0.000039	1.392490
C	-0.696462	-1.195699	1.554154
C	0.695995	-1.195656	1.554341
C	1.401394	0.000036	1.392862
C	2.720796	0.000029	0.673372
C	-2.721078	-0.000052	0.672650
C	2.720975	-0.000095	-0.673000
C	-2.720899	0.000084	-0.673722
C	1.401764	-0.000054	-1.392840
C	0.696337	-1.195701	-1.554506
C	-0.696121	-1.195632	-1.554694
C	-1.401498	0.000072	-1.393212
C	-0.696028	1.195725	-1.554645
C	0.696430	1.195656	-1.554463
C	0.695946	1.195701	1.554298
H	-1.230089	2.146070	1.483406
H	-1.229983	-2.146146	1.483489
H	1.229591	-2.146070	1.483807
H	1.229840	-2.146157	-1.483843
H	-1.229734	-2.146037	-1.484161
H	-1.229549	2.146179	-1.484087

H	1.230025	2.146058	-1.483752
H	1.229485	2.146145	1.483741
H	3.667317	0.000053	1.220742
H	3.667642	-0.000131	-1.220119
H	-3.667745	-0.000076	1.219769
H	-3.667421	0.000130	-1.221092

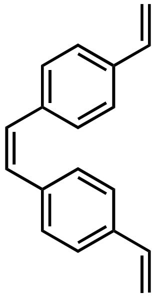

34

16, E = -695.029650556 Ha

C	0.105100	0.762402	1.676013
C	-1.044904	-0.029254	1.805082
C	-1.145288	-0.871664	2.915669
C	-0.114622	-0.951291	3.844874
C	1.045004	-0.184288	3.702227
C	3.668919	-0.345101	-3.796317
C	-2.169220	0.056064	0.844974
C	2.482196	0.226320	-4.001080
C	-2.111288	0.207968	-0.485290
C	1.324874	0.189599	-3.082471
C	1.255448	-0.681131	-1.986663
C	0.159243	-0.675180	-1.138598
C	-0.912311	0.202902	-1.354543
C	-0.860141	1.047188	-2.467016
C	0.242904	1.043807	-3.312779
C	1.128987	0.685876	2.606988
H	0.182901	1.454514	0.836209
H	-2.044121	-1.478045	3.047747
H	-0.207533	-1.623415	4.701032
H	2.064899	-1.390139	-1.806380
H	0.117401	-1.368690	-0.297387
H	-1.696185	1.721061	-2.666938
H	0.269452	1.718513	-4.171532
H	2.002583	1.329409	2.492523
H	2.323338	0.796380	-4.921701
H	-3.164210	0.004480	1.297442
H	-3.060959	0.335205	-1.013807
H	3.899402	-0.905497	-2.888398
H	4.465936	-0.262501	-4.535356
C	2.120827	-0.307886	4.708165

C	3.360645	0.169126	4.598289
H	1.846925	-0.861101	5.611867
H	3.703996	0.707189	3.712805
H	4.088793	0.027193	5.396971

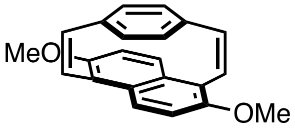

42

17 E = -999.1626655 Ha, ΔH = 225.799 kcal/mol, ΔS = 136.744 cal/mol•K

C	0.721727	-1.185767	1.983445
C	-0.668588	-1.235818	1.853405
C	1.382748	0.040264	1.974083
H	1.302913	-2.108720	1.924499
C	0.668165	1.235962	1.853439
H	2.473415	0.069614	1.905868
C	-0.722148	1.185913	1.983363
C	1.336506	2.436605	1.253723
C	-1.383171	-0.040125	1.973954
C	-1.336796	-2.436530	1.253682
H	-1.303343	2.108856	1.924335
H	-2.473834	-0.069461	1.905641
C	-0.148737	-0.691096	-1.028094
C	0.148870	0.690924	-1.028099
C	-2.514987	-0.200051	-0.916623
C	-0.931804	1.607938	-1.127637
C	-2.229786	1.169922	-1.164391
C	-1.494634	-1.116989	-0.792872
C	-1.729387	-2.386298	-0.031199
H	-0.713120	2.675813	-1.179054
C	0.931947	-1.608105	-1.127379
O	-3.776270	-0.654545	-0.652948
C	1.494707	1.116847	-0.792676
H	-3.038329	1.891321	-1.274574
C	2.229938	-1.170078	-1.163946
C	2.515098	0.199903	-0.916238
H	0.713293	-2.675989	-1.178733
H	3.038501	-1.891492	-1.273891
C	1.729356	2.386207	-0.031075
O	3.776336	0.654441	-0.652430
C	-4.825424	0.288910	-0.598486
C	4.825676	-0.288850	-0.598799
H	-2.241562	-3.235220	-0.490476
H	-1.479124	-3.348550	1.839747
H	2.241743	3.235019	-0.490324
H	1.478807	3.348671	1.839719
H	-5.726161	-0.269479	-0.321677

H	-4.987370	0.772573	-1.575528
H	-4.628649	1.063624	0.161461
H	5.726465	0.269616	-0.322315
H	4.987172	-0.772212	-1.576062
H	4.629452	-1.063810	0.161041

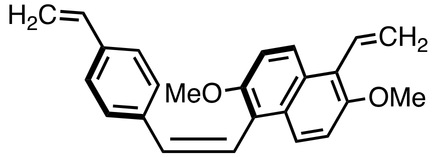

48

18, E = -1077.753669 Ha, ΔH = 262.827 kcal/mol, ΔS = 165.306 cal/mol•K

C	-0.505772	0.290496	2.395709
C	-1.464887	-0.730494	2.356546
C	-0.739712	1.459179	3.105903
H	0.440089	0.152915	1.869182
C	-1.941589	1.653107	3.799999
H	0.034162	2.227923	3.137517
C	-2.882580	0.618827	3.785663
C	-2.240696	2.891464	4.549691
C	-2.646238	-0.556633	3.081736
C	-1.250756	-1.973300	1.582446
H	-3.817804	0.742071	4.337156
H	-3.397425	-1.349492	3.077613
C	1.024531	-0.883663	-1.048970
C	1.445659	0.195209	-1.871785
C	-1.151922	0.140368	-0.741377
C	0.532513	1.267472	-2.067129
C	-0.722186	1.243156	-1.520956
C	-0.288168	-0.906950	-0.481958
C	-0.715052	-2.052017	0.358882
H	0.841558	2.140867	-2.639976
C	1.959469	-1.913136	-0.769787
O	-2.413420	0.071204	-0.236300
C	2.757639	0.211163	-2.448661
H	-1.401265	2.078055	-1.691608
C	3.222613	-1.884249	-1.295555
C	3.629259	-0.823668	-2.144362
H	1.673418	-2.729164	-0.105604
H	3.917579	-2.686440	-1.053490
C	-1.566317	4.039163	4.478572
O	4.876187	-0.788207	-2.692657
C	-3.065287	1.290328	0.073544
C	5.717426	-1.911208	-2.530692
H	-0.599204	-3.045748	-0.085124
H	-1.590812	-2.898242	2.057449
H	-3.113638	2.833555	5.207444

H	-3.921806	1.027365	0.704318
H	-3.428768	1.801017	-0.833128
H	-2.394915	1.959357	0.637123
H	6.615686	-1.705646	-3.122777
H	5.237745	-2.830005	-2.904668
H	6.005646	-2.053104	-1.476445
H	-1.864352	4.902523	5.073589
H	-0.701544	4.169848	3.825349
C	3.119316	1.289178	-3.396004
C	4.313051	1.866268	-3.536211
H	2.306296	1.624705	-4.046289
H	5.167672	1.582570	-2.924275
H	4.462561	2.650792	-4.279623

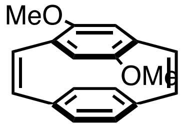

36

19, E = -845.573681742 Ha

C	-8.129341	3.196081	-1.381883
H	-9.720807	4.325975	-2.300810
C	-6.789101	1.340335	-3.669815
C	-8.646930	4.255999	-2.128490
C	-6.753518	2.942654	-1.408332
C	-5.969279	2.148470	-4.462705
C	-7.888349	3.453401	-5.148260
C	-7.812036	5.067684	-2.896294
C	-5.913184	3.914773	-1.950872
C	-6.430668	4.978622	-2.691981
H	-4.844000	3.704861	-1.975003
O	-8.907301	2.238824	-0.794481
C	-6.513832	3.201614	-5.194112
C	-8.169677	1.410254	-3.879341
C	-8.714433	2.458225	-4.618108
C	-6.265964	0.754700	-2.388935
C	-8.382872	4.867138	-5.267005
C	-8.329842	5.655220	-4.178452
C	-6.264149	1.527273	-1.288054
O	-5.656422	5.829217	-3.429330
H	-8.750446	5.264700	-6.216703
H	-8.642454	6.700581	-4.237811
H	-5.920780	-0.281159	-2.334709
H	-5.928763	1.122605	-0.330040
H	-9.797458	2.606045	-4.619593
H	-8.829232	0.757929	-3.301652
C	-4.262071	5.618119	-3.417693
H	-3.825606	6.397809	-4.051295

H	-4.004036	4.626390	-3.828441
H	-3.850309	5.703206	-2.398574
C	-10.305008	2.351165	-0.944993
H	-10.743781	1.507696	-0.401005
H	-10.681386	3.295667	-0.518798
H	-10.599034	2.293437	-2.007555
H	-5.851231	3.946210	-5.642234
H	-4.883809	2.074371	-4.357541

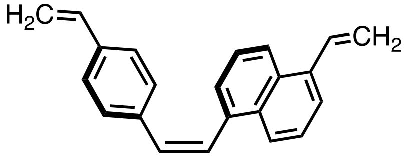

42

20, E = -924.184927311 Ha

C	-7.387627	3.173868	-0.995485
H	-8.378596	3.051036	-2.909533
C	-7.688813	0.029755	-5.519888
C	-7.642443	3.574134	-2.303032
C	-6.457745	3.875706	-0.210126
C	-6.621395	0.682849	-4.887083
C	-7.589903	2.852461	-5.378394
C	-6.962744	4.650198	-2.877354
C	-5.811908	4.980720	-0.770077
C	-6.048978	5.365205	-2.087071
H	-5.124876	5.548210	-0.144297
O	-7.999751	2.104347	-0.409154
C	-6.573006	2.066296	-4.817558
C	-8.683663	0.815481	-6.107729
C	-8.633526	2.204065	-6.043424
C	-7.555059	4.333350	-5.313068
C	-7.232133	5.096776	-4.259290
C	-6.204292	3.440733	1.177622
O	-5.439900	6.429077	-2.688862
H	-7.817168	4.846595	-6.243061
H	-7.173476	6.176832	-4.410797
H	-9.423684	2.798527	-6.507325
H	-9.514508	0.326798	-6.621991
C	-4.543620	7.195132	-1.912773
H	-5.047868	7.636946	-1.037671
H	-4.177498	7.995741	-2.564245
H	-3.691407	6.585544	-1.570203
C	-8.871320	1.333375	-1.212959
H	-9.196667	0.491003	-0.593328
H	-9.752006	1.918871	-1.523578
H	-8.354527	0.954703	-2.110647
H	-5.734878	2.560524	-4.323574

H	-5.805590	0.097909	-4.459225
C	-7.790615	-1.442903	-5.596839
C	-5.118855	3.741873	1.891389
H	-6.980719	2.811938	1.616581
H	-4.293020	4.326690	1.480396
H	-5.008514	3.394250	2.918725
C	-7.070165	-2.319582	-4.897305
H	-8.548144	-1.818186	-6.291906
H	-6.317004	-2.009790	-4.170383
H	-7.216518	-3.392516	-5.022310

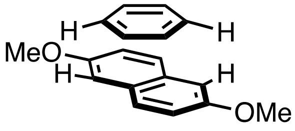

3

38

21, E = -846.7742819 Ha

C	-3.42448	2.76006	2.14839
C	-3.92319	3.85476	2.85896
C	-2.09060	2.72925	1.75197
H	-4.11092	2.01356	1.75817
C	-1.22694	3.78257	2.06731
H	-1.75451	1.95949	1.06285
C	-1.66421	4.72548	3.00097
C	-3.00099	4.76348	3.38681
H	-1.00327	5.53503	3.29851
H	-3.36316	5.59754	3.98140
C	-3.90990	5.26764	0.19308
C	-2.53731	5.57410	0.02379
C	-4.17078	7.16247	1.67373
C	-2.05516	6.78663	0.57761
C	-2.87693	7.60908	1.30387
C	-4.66594	5.97189	1.18265
H	-1.01938	7.06680	0.41344
C	-4.44157	4.17760	-0.54067
O	-4.93732	7.79555	2.60661
C	-1.67314	4.60274	-0.57312
H	-2.48929	8.54846	1.68144
C	-3.64110	3.40790	-1.34445
C	-2.23366	3.57381	-1.30185
H	-5.50221	3.95879	-0.46686
H	-4.08422	2.60531	-1.92302
O	-1.36274	2.68341	-1.85629
C	-4.39251	8.90157	3.28667
H	-4.20642	9.74636	2.61077
H	-3.45903	8.64098	3.80293
H	-5.13980	9.19717	4.02456

C	-1.87976	1.51143	-2.44090
H	-2.49500	1.73137	-3.32308
H	-2.47308	0.92808	-1.72432
H	-1.01397	0.92451	-2.75107
H	-0.61098	4.61447	-0.13666
H	-5.51913	5.40846	1.78242
H	-5.07366	4.15719	2.76185
H	-0.29899	4.01810	1.34114

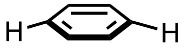

12

23, E = -232.1056674 Ha

C	-3.42448	2.76006	2.14839
C	-3.92319	3.85476	2.85896
C	-2.09060	2.72925	1.75197
H	-4.11092	2.01356	1.75817
C	-1.22694	3.78257	2.06731
H	-1.75451	1.95949	1.06285
C	-1.66421	4.72548	3.00097
C	-3.00099	4.76348	3.38681
H	-1.00327	5.53503	3.29851
H	-3.36316	5.59754	3.98140
H	-5.07366	4.15719	2.76185
H	-0.29899	4.01810	1.34114

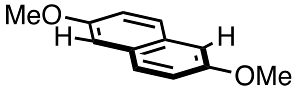

26

24, E = -614.6804676 Ha

C	-3.90990	5.26764	0.19308
C	-2.53731	5.57410	0.02379
C	-4.17078	7.16247	1.67373
C	-2.05516	6.78663	0.57761
C	-2.87693	7.60908	1.30387
C	-4.66594	5.97189	1.18265
H	-1.01938	7.06680	0.41344
C	-4.44157	4.17760	-0.54067
O	-4.93732	7.79555	2.60661
C	-1.67314	4.60274	-0.57312
H	-2.48929	8.54846	1.68144
C	-3.64110	3.40790	-1.34445
C	-2.23366	3.57381	-1.30185
H	-5.50221	3.95879	-0.46686
H	-4.08422	2.60531	-1.92302
O	-1.36274	2.68341	-1.85629
C	-4.39251	8.90157	3.28667
H	-4.20642	9.74636	2.61077

H	-3.45903	8.64098	3.80293
H	-5.13980	9.19717	4.02456
C	-1.87976	1.51143	-2.44090
H	-2.49500	1.73137	-3.32308
H	-2.47308	0.92808	-1.72432
H	-1.01397	0.92451	-2.75107
H	-0.61098	4.61447	-0.13666
H	-5.51913	5.40846	1.78242

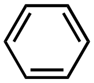

12

25, E = -232.1359957 Ha

C	-3.458074	2.834541	2.021073
C	-3.913784	3.860992	2.845182
C	-2.122313	2.798413	1.627141
H	-4.147420	2.058773	1.684029
C	-1.242171	3.788761	2.057357
H	-1.765548	1.994332	0.981548
C	-1.697719	4.814852	2.881972
C	-3.033517	4.850998	3.275888
H	-1.008282	5.590374	3.219399
H	-3.390168	5.654872	3.921797
H	-4.960128	3.889117	3.153877
H	-0.195796	3.760485	1.749066

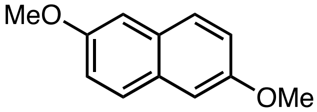

26

26, E = -614.7147816 Ha

C	-3.872718	5.171808	0.348564
C	-2.495653	5.473850	0.173311
C	-4.148206	7.126955	1.755806
C	-1.976525	6.628645	0.816057
C	-2.771122	7.437212	1.586326
C	-4.680853	6.016638	1.147241
H	-0.919347	6.869986	0.688130
C	-4.391785	4.017016	-0.294171
O	-4.995235	7.886980	2.507469
C	-1.687496	4.629146	-0.625435
H	-2.339451	8.315122	2.064801
C	-3.597150	3.208541	-1.064548
C	-2.220169	3.518906	-1.234166
H	-5.448900	3.775455	-0.166160
H	-4.028802	2.330577	-1.542968
O	-1.373248	2.759066	-1.986135
C	-4.471863	9.033747	3.143518

H	-4.076461	9.756617	2.411178
H	-3.676360	8.770524	3.859821
H	-5.306373	9.491378	3.685499
C	-1.896899	1.612432	-2.622189
H	-2.692952	1.875764	-3.337846
H	-2.291686	0.889290	-1.889788
H	-1.062706	1.155102	-3.164911
H	-0.628798	4.849302	-0.768350
H	-5.739571	5.796521	1.290225

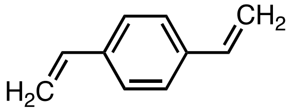

20

27, E = -386.8593816 Ha

C	-3.247075	2.539047	2.103102
C	-3.829262	3.432982	3.007815
C	-1.965265	2.756268	1.618849
H	-3.801619	1.660515	1.770183
C	-1.214078	3.869552	2.014570
H	-1.528515	2.046150	0.912703
C	-1.796427	4.763711	2.918960
C	-3.078253	4.546515	3.403179
H	-1.242267	5.642769	3.251124
H	-3.515165	5.256858	4.108996
C	0.145863	4.048803	1.464927
C	0.986459	5.048445	1.730762
H	0.466113	3.264554	0.771981
H	0.734410	5.864180	2.410636
H	1.973194	5.086449	1.269285
C	-5.189040	3.253482	3.557782
C	-6.025860	2.248710	3.299483
H	-5.512446	4.041993	4.244397
H	-5.770129	1.428051	2.626947
H	-7.012898	2.211055	3.760341

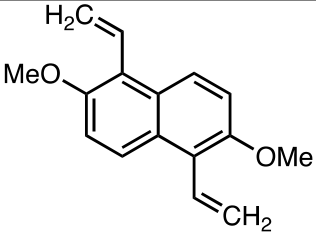

34

28, E = -769.4313251 Ha

C	-3.786006	5.086137	0.503362
C	-2.440376	5.438863	0.214076
C	-4.063141	7.072963	1.865294
C	-1.950999	6.657242	0.754288

C	-2.734446	7.450647	1.547287
C	-4.598314	5.898063	1.362053
H	-0.944360	6.991783	0.507402
C	-4.305511	3.918535	-0.115157
O	-4.849355	7.839954	2.671821
C	-1.618483	4.610819	-0.619738
H	-2.326600	8.384716	1.929839
C	-3.533384	3.144213	-0.937580
C	-2.179459	3.479405	-1.190065
H	-5.349581	3.647015	0.034473
H	-3.973688	2.264779	-1.404367
O	-1.390034	2.707072	-1.988318
C	-4.267320	8.950087	3.322883
H	-3.963789	9.728418	2.604032
H	-3.395128	8.650450	3.925952
H	-5.041558	9.355231	3.983297
C	-1.895880	1.468681	-2.441696
H	-2.742668	1.605469	-3.133732
H	-2.211815	0.831281	-1.600208
H	-1.072776	0.981313	-2.975273
C	-5.951986	5.430579	1.735580
C	-7.035214	6.184272	1.925902
H	-6.046652	4.349882	1.875792
H	-7.014921	7.266176	1.805491
H	-7.983545	5.725710	2.210342
C	-0.188464	4.949724	-0.794929
C	0.543639	4.787582	-1.897379
H	0.301064	5.365152	0.090680
H	0.123740	4.378872	-2.815019
H	1.598350	5.066926	-1.905475

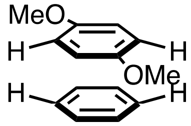

32

29, E = -693.213890723 Ha

C	-8.12934	3.19608	-1.38188
H	-9.72081	4.32597	-2.30081
C	-6.78910	1.34034	-3.66981
C	-8.64693	4.25600	-2.12849
C	-6.75352	2.94265	-1.40833
C	-5.96928	2.14847	-4.46270
C	-7.88835	3.45340	-5.14826
C	-7.81204	5.06768	-2.89629
C	-5.91318	3.91477	-1.95087
C	-6.43067	4.97862	-2.69198
H	-4.84400	3.70486	-1.97500

O	-8.90730	2.23882	-0.79448
C	-6.51383	3.20161	-5.19411
C	-8.16968	1.41025	-3.87934
C	-8.71443	2.45823	-4.61811
H	-6.40557	0.91569	-2.74273
H	-8.24493	4.47506	-5.23326
H	-8.18662	5.49170	-3.82440
H	-6.40357	1.92383	-1.32177
O	-5.65642	5.82922	-3.42933
H	-9.79746	2.60604	-4.61959
H	-8.82923	0.75793	-3.30165
C	-4.26207	5.61812	-3.41769
H	-3.82561	6.39781	-4.05129
H	-4.00404	4.62639	-3.82844
H	-3.85031	5.70321	-2.39857
C	-10.30501	2.35116	-0.94499
H	-10.74378	1.50770	-0.40101
H	-10.68139	3.29567	-0.51880
H	-10.59903	2.29344	-2.00755
H	-5.85123	3.94621	-5.64223
H	-4.88381	2.07437	-4.35754

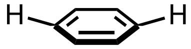

12

30, E = -232.112227783 Ha

C	-6.78910	1.34034	-3.66981
C	-5.96928	2.14847	-4.46270
C	-7.88835	3.45340	-5.14826
C	-6.51383	3.20161	-5.19411
C	-8.16968	1.41025	-3.87934
C	-8.71443	2.45823	-4.61811
H	-6.40557	0.91569	-2.74273
H	-8.24493	4.47506	-5.23326
H	-9.79746	2.60604	-4.61959
H	-8.82923	0.75793	-3.30165
H	-5.85123	3.94621	-5.64223
H	-4.88381	2.07437	-4.35754

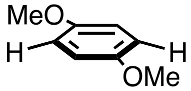

20

31, E = -461.117498404 Ha

C	-8.12934	3.19608	-1.38188
H	-9.72081	4.32597	-2.30081
C	-8.64693	4.25600	-2.12849
C	-6.75352	2.94265	-1.40833

C	-7.81204	5.06768	-2.89629
C	-5.91318	3.91477	-1.95087
C	-6.43067	4.97862	-2.69198
H	-4.84400	3.70486	-1.97500
O	-8.90730	2.23882	-0.79448
H	-8.18662	5.49170	-3.82440
H	-6.40357	1.92383	-1.32177
O	-5.65642	5.82922	-3.42933
C	-4.26207	5.61812	-3.41769
H	-3.82561	6.39781	-4.05129
H	-4.00404	4.62639	-3.82844
H	-3.85031	5.70321	-2.39857
C	-10.30501	2.35116	-0.94499
H	-10.74378	1.50770	-0.40101
H	-10.68139	3.29567	-0.51880
H	-10.59903	2.29344	-2.00755

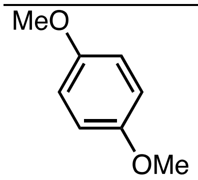

20

32, E = -461.141001576 Ha

C	-8.027930	2.502224	-1.315543
H	-9.484346	4.108473	-1.410644
C	-8.454756	3.803512	-1.592225
C	-6.701841	2.151359	-1.561982
C	-7.555869	4.730061	-2.108089
C	-5.802954	3.077907	-2.077850
C	-6.229779	4.379197	-2.354526
H	-4.773367	2.772943	-2.259442
O	-8.830249	1.520127	-0.808642
O	-5.427462	5.361291	-2.861435
C	-4.081284	5.027123	-3.118064
H	-3.607232	5.931409	-3.514565
H	-4.003274	4.218410	-3.863909
H	-3.561256	4.718072	-2.195870
C	-10.176363	1.854367	-0.551774
H	-10.650383	0.950118	-0.155150
H	-10.254194	2.663113	0.194052
H	-10.696552	2.163405	-1.473883
H	-6.388665	1.131317	-1.339428
H	-7.869044	5.750103	-2.330642

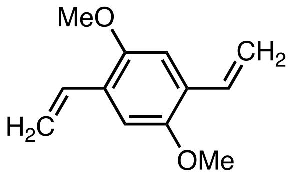

28

33, E = -615.866023495 Ha

C	-8.021817	2.503340	-1.317683
H	-9.474755	4.088639	-1.407322
C	-8.442396	3.800318	-1.594719
C	-6.689557	2.123657	-1.557148
C	-7.568246	4.757654	-2.113282
C	-5.815392	3.081010	-2.075654
C	-6.235975	4.377984	-2.352705
H	-4.782992	2.792736	-2.262896
O	-8.847527	1.540786	-0.810417
C	-5.043552	0.237452	-1.429382
C	-9.214682	6.643371	-2.242675
C	-7.991937	6.139348	-2.414876
C	-6.265864	0.741966	-1.255546
O	-5.410258	5.340544	-2.859948
H	-7.205478	6.780243	-2.814526
H	-7.051944	0.101498	-0.854468
C	-4.066390	4.992111	-3.111572
H	-3.583911	5.891510	-3.508804
H	-3.995724	4.181590	-3.855449
H	-3.553594	4.681547	-2.186433
C	-10.191278	1.889353	-0.558346
H	-10.673706	0.990012	-0.160922
H	-10.261612	2.699909	0.185523
H	-10.704362	2.199928	-1.483321
H	-4.209258	0.819391	-1.825864
H	-4.830053	-0.800750	-1.174836
H	-9.428123	7.681640	-2.497000
H	-10.049427	6.060923	-1.847892

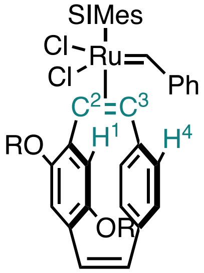

101

34, E = -2163.84554728 Ha

Ru	-0.629324	0.614969	-0.307788

Cl	-0.946849	1.471029	-2.645838
Cl	-0.168330	0.014229	2.080969
N	-3.520420	1.156016	0.675376
N	-2.072329	2.807937	0.840320
C	-2.251120	1.530660	0.417818
C	-4.300286	2.223790	1.355098
H	-4.597778	1.889591	2.357967
H	-5.206688	2.454364	0.780026
C	-3.297247	3.413446	1.403154
H	-3.620324	4.262168	0.783525
H	-3.116548	3.771155	2.425047
C	-4.122179	-0.096409	0.310918
C	-4.211088	-1.132839	1.258129
C	-4.797530	-2.345036	0.860376
H	-4.844169	-3.169053	1.576519
C	-5.293316	-2.531016	-0.439398
C	-5.201206	-1.468764	-1.356604
H	-5.582435	-1.599493	-2.372073
C	-4.622451	-0.242158	-0.999681
C	-3.663028	-0.957309	2.656987
H	-2.686472	-0.452953	2.638979
H	-3.533923	-1.933601	3.143253
H	-4.349264	-0.362287	3.281054
C	-5.919722	-3.849468	-0.844607
H	-7.011553	-3.821892	-0.710664
H	-5.523959	-4.674342	-0.236831
H	-5.717773	-4.075029	-1.901050
C	-4.513401	0.896929	-1.990149
H	-4.943704	1.824263	-1.579090
H	-5.044826	0.659413	-2.920411
H	-3.461756	1.109354	-2.241261
C	-0.833736	3.496053	0.568241
C	-0.678691	4.135206	-0.682040
C	0.593442	4.620258	-1.025354
H	0.734918	5.075412	-2.007610
C	1.683505	4.510056	-0.145628
C	1.463179	3.978548	1.136570
H	2.289103	3.937343	1.850932
C	0.205785	3.490778	1.526665
C	-1.858681	4.347877	-1.606136
H	-2.357211	5.300002	-1.359202
H	-2.587712	3.530764	-1.530086
H	-1.530430	4.383440	-2.650699
C	3.072415	4.954178	-0.554919
H	3.489947	5.668352	0.168786
H	3.066816	5.429191	-1.544233

H	3.749977	4.087577	-0.592162
C	-0.034169	3.083305	2.964558
H	0.806858	2.497301	3.356154
H	-0.931561	2.465007	3.070299
H	-0.140545	3.990210	3.581310
C	-1.474216	-0.925373	-0.882122
H	-1.908189	-0.784989	-1.890164
C	-1.649843	-2.316956	-0.430270
C	-2.206205	-3.205551	-1.382970
H	-2.464437	-2.824112	-2.373569
C	-2.428885	-4.552534	-1.074014
H	-2.849447	-5.222281	-1.824565
C	-2.110722	-5.032416	0.205580
H	-2.280636	-6.081166	0.453521
C	-1.562409	-4.161344	1.163930
H	-1.310540	-4.534838	2.157290
C	-1.327574	-2.818508	0.851029
H	-0.911973	-2.142935	1.595391
C	4.614357	-2.425443	-1.176555
C	4.602038	-2.635428	0.212797
C	3.369414	-2.543524	0.883430
C	2.262350	-1.959828	0.245870
C	2.362419	-1.506692	-1.074567
C	1.552271	-0.325015	-1.531371
C	5.866685	-2.530977	1.029627
C	1.860244	0.907133	-0.990816
C	6.239497	-1.301906	1.472628
C	3.091770	1.002771	-0.117913
C	3.115675	0.797033	1.272675
C	4.254526	0.232226	1.877986
C	5.373988	-0.122185	1.100520
C	5.450553	0.391591	-0.207914
C	4.316201	0.945137	-0.815866
C	3.496336	-1.873291	-1.823583
H	2.193805	0.901469	1.845912
H	6.340000	0.193530	-0.809589
H	0.891593	-0.383829	-2.398163
H	1.413406	1.808768	-1.431178
H	6.441262	-3.419293	1.297644
H	7.126475	-1.182238	2.098415
O	3.514143	-1.439141	-3.150196
O	3.382641	-2.849039	2.239136
H	1.388274	-1.663136	0.827665
H	5.564685	-2.511610	-1.703222
C	4.734490	-1.600662	-3.902224
C	2.173669	-2.604982	2.997707

H	1.328042	-3.176574	2.578745
H	1.910651	-1.536123	2.998867
H	2.384514	-2.946627	4.015296
H	5.559430	-1.026559	-3.446148
H	4.526417	-1.213007	-4.903281
H	5.022165	-2.662080	-3.968136
H	4.329265	1.161053	-1.887419
H	4.196901	-0.098109	2.916711

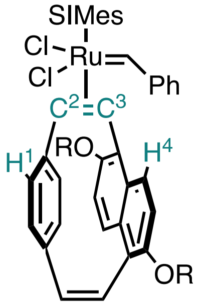

107

35, E = -2317.38042431 Ha

Ru	0.932235	-0.602652	-0.257443
Cl	1.343897	-1.813927	-2.409451
Cl	0.399161	0.325172	2.006246
N	3.761813	-1.019822	0.912286
N	2.293335	-2.627991	1.243563
C	2.500890	-1.424411	0.651500
C	4.499467	-1.979218	1.775085
H	4.753941	-1.502342	2.730995
H	5.429075	-2.292749	1.282037
C	3.484809	-3.144993	1.948662
H	3.827326	-4.077404	1.477582
H	3.250047	-3.347303	3.001690
C	4.406275	0.156352	0.398211
C	4.477614	1.319609	1.186394
C	5.115887	2.447368	0.645673
H	5.149535	3.367492	1.234166
C	5.681306	2.428113	-0.638637
C	5.608493	1.242505	-1.391872
H	6.045332	1.212759	-2.392810
C	4.979277	0.094517	-0.889342
C	3.861409	1.364551	2.566921
H	2.883310	0.863556	2.581507
H	3.716427	2.405001	2.887564
H	4.513948	0.875208	3.308135
C	6.360662	3.659926	-1.200616
H	7.443009	3.634531	-1.003959
H	5.954419	4.573165	-0.745199
H	6.219825	3.727701	-2.288421

C	4.892750	-1.178556	-1.703340
H	5.289227	-2.038470	-1.139767
H	5.468025	-1.088727	-2.633476
H	3.849519	-1.416149	-1.967303
C	1.076316	-3.370981	1.027237
C	0.990362	-4.204852	-0.110315
C	-0.251183	-4.785825	-0.413485
H	-0.340393	-5.394782	-1.315247
C	-1.376924	-4.583212	0.403019
C	-1.223944	-3.845597	1.588805
H	-2.078244	-3.719992	2.258502
C	0.000220	-3.252057	1.936264
C	2.209428	-4.521649	-0.950346
H	2.700347	-5.430561	-0.564370
H	2.931605	-3.695225	-0.949986
H	1.924864	-4.692600	-1.994444
C	-2.731823	-5.146553	0.029174
H	-3.135392	-5.775019	0.835721
H	-2.676817	-5.751517	-0.884835
H	-3.446565	-4.327331	-0.140965
C	0.163111	-2.607679	3.296338
H	-0.694153	-1.964913	3.533778
H	1.057421	-1.978410	3.348120
H	0.225486	-3.397174	4.062309
C	1.866757	0.801993	-1.004489
H	2.381324	0.496411	-1.934200
C	2.030393	2.248725	-0.767315
C	2.664472	2.973948	-1.805712
H	2.980846	2.440998	-2.705255
C	2.890930	4.350703	-1.693561
H	3.372733	4.892694	-2.507829
C	2.498914	5.025241	-0.527063
H	2.672207	6.098176	-0.432184
C	1.871405	4.317915	0.513892
H	1.559432	4.842780	1.417312
C	1.630854	2.945008	0.396211
H	1.151793	2.396713	1.205501
C	-1.619052	-1.209228	-0.901403
C	-2.866862	-1.216095	-0.055825
C	-4.099253	-1.406392	-0.712878
H	-4.101653	-1.804980	-1.730960
C	-5.291979	-0.926600	-0.148633
H	-6.211827	-0.953140	-0.735105
C	-5.262024	-0.229389	1.072999
C	-4.072069	-0.261130	1.825031
H	-4.048944	0.237452	2.796977

C	-6.311318	0.798552	1.405391
H	-7.304663	0.495609	1.744525
C	-5.998570	2.112565	1.264459
H	-6.711092	2.892442	1.538389
C	-4.615067	2.459128	0.783265
C	-4.214496	2.040503	-0.534008
C	-5.148498	1.870512	-1.601140
C	-4.756879	1.314069	-2.802417
C	-3.454979	0.734282	-2.918097
O	-3.100142	-0.097874	-3.980197
C	-4.029129	-0.267601	-5.072470
H	-4.969217	-0.728832	-4.728745
H	-3.533342	-0.934601	-5.782970
H	-4.249031	0.694456	-5.561617
C	-2.533422	0.850389	-1.891767
H	-5.471420	1.231135	-3.620595
H	-6.179117	2.196182	-1.455321
C	-2.861983	1.671679	-0.760886
C	-1.885485	2.034978	0.209552
H	-0.838232	1.801040	0.021405
C	-2.249244	2.666099	1.378902
H	-1.476471	2.932687	2.099004
C	-3.635207	2.810851	1.695629
O	-4.071738	3.167760	2.971854
C	-3.081665	3.358146	4.008201
H	-2.455200	2.459587	4.128305
H	-2.438451	4.224946	3.789430
H	-3.643955	3.543696	4.927829
C	-2.880255	-0.755306	1.271228
H	-1.937455	-0.623936	1.804041
C	-1.448098	-0.188107	-1.802079
H	-0.674548	-0.257905	-2.569610
H	-0.971707	-2.097979	-0.953957

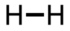

2

Hydrogen, E = -1.165495651 Ha

H	-0.775208	0.899575	-0.000000
H	-0.023292	0.884725	0.000000

12. References

[1] A. de Meijere, S. I. Kozhushkov, K. Rauch, H. Schill, S. P. Verevkin, M. Kümmerlin, H.-D. Beckhaus, C. Rüchardt, D. S. Yufit, *J. Am. Chem. Soc.* 2003, **125**, 15110.
[2] Gaussian 16, Revision A.03, M. J. Frisch, G. W. Trucks, H. B. Schlegel, G. E. Scuseria, M. A. Robb, J. R. Cheeseman, G. Scalmani, V. Barone, G. A. Petersson, H. Nakatsuji, X. Li, M. Caricato, A. V. Marenich, J. Bloino, B. G. Janesko, R. Gomperts, B. Mennucci, H. P. Hratchian, J. V. Ortiz, A. F. Izmaylov, J. L. Sonnenberg, D. Williams-Young, F. Ding, F. Lipparini, F. Egidi, J. Goings, B. Peng, A. Petrone, T. Henderson, D. Ranasinghe, V. G. Zakrzewski, J. Gao, N. Rega, G. Zheng, W. Liang, M. Hada, M. Ehara, K. Toyota, R. Fukuda, J. Hasegawa, M. Ishida, T. Nakajima, Y. Honda, O. Kitao, H. Nakai, T. Vreven, K. Throssell, J. A. Montgomery, Jr., J. E. Peralta, F. Ogliaro, M. J. Bearpark, J. J. Heyd, E. N. Brothers, K. N. Kudin, V. N. Staroverov, T. A. Keith, R. Kobayashi, J. Normand, K. Raghavachari, A. P. Rendell, J. C. Burant, S. S. Iyengar, J. Tomasi, M. Cossi, J. M. Millam, M. Klene, C. Adamo, R. Cammi, J. W. Ochterski, R. L. Martin, K. Morokuma, O. Farkas, J. B. Foresman, and D. J. Fox, Gaussian, Inc., Wallingford CT, 2016.
[3] M. D. Hanwell, D. E. Curtis, D. C. Lonie, T. Vandermeersch, E. Zurek, G. R. Hutchinson. *J. Cheminform.* **2012**, *4*, 17.
[4] A. K. Rappé, C. J. Casewit, K. S. Colwell, W. A. Goddard III, W. M. Skiff, *J. Am. Chem. Soc.* **1992**, *114*, 10024–10035.
[5] R. Krishnan, J. S. Binkley, R. Seeger, J. A. Pople, *J. Chem. Phys*. **1980**, *72*, 650-654.
[6] A. D. McLean, G. S. Chandler, *Chem. Phys.* **1980**, *72*, 5639-5648.
[7] R. Peverati, D. G. Truhlar, *J. Phys. Chem. Lett.* **2011**, *2*, 2810–2817.
[8] A. Mann, M. D. Hannigan, M. Weck, *Macromol. Chem. Phys.* **2023**, *224*, 2200397.
[9] P. v. R. Schleyer, J. E. Williams, K. R. Blanchard, *J. Am. Chem. Soc.* **1970**, *92*, 2377–2386.
[10] S. Grimme, C. Mück-Lichtenfeld, *Isr. J. Chem.* **2012**, *52*, 180–192.

13. NMR Spectra

^1H NMR of **4** (400 MHz, $CDCl_3$)

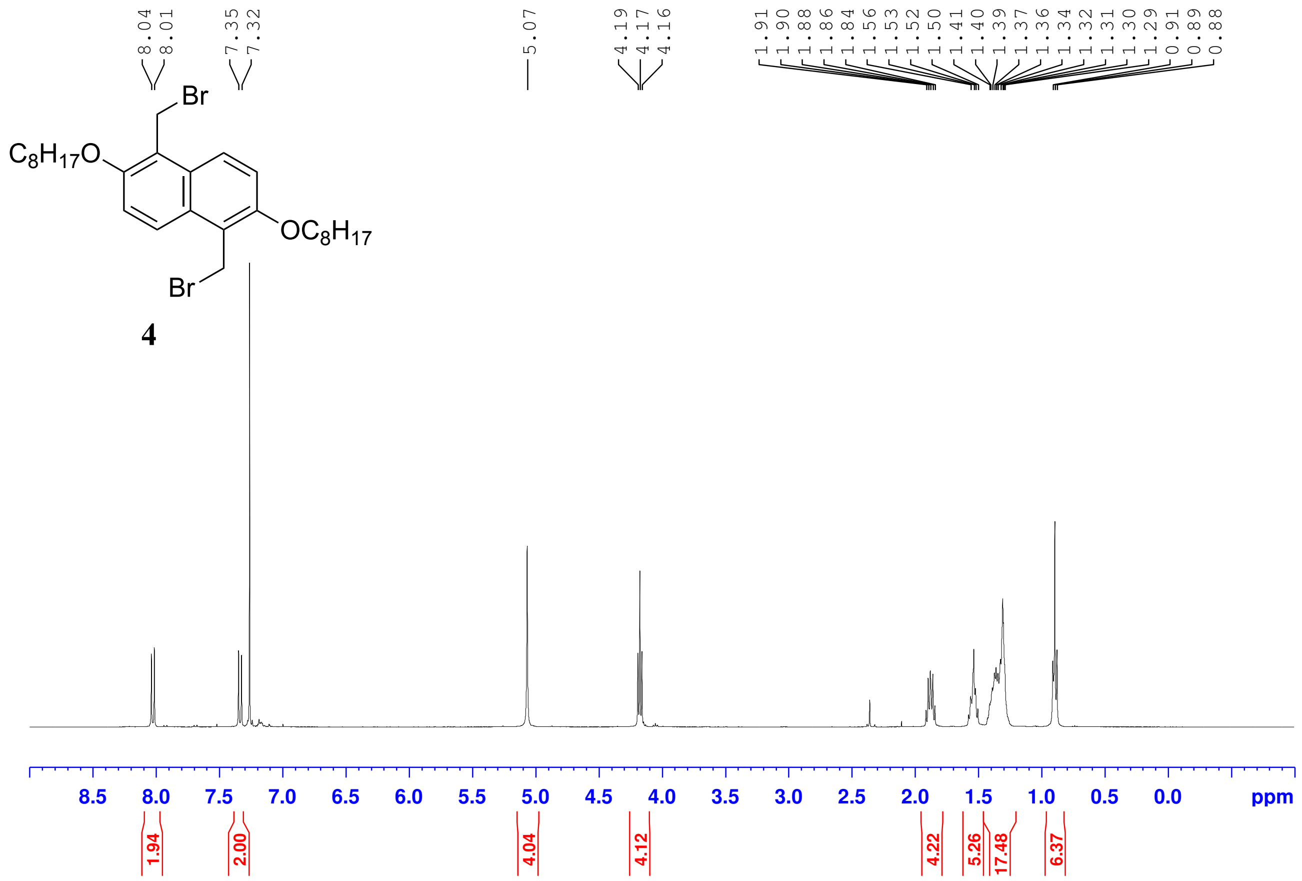

^1H NMR of **5** (400 MHz, $CDCl_3$)

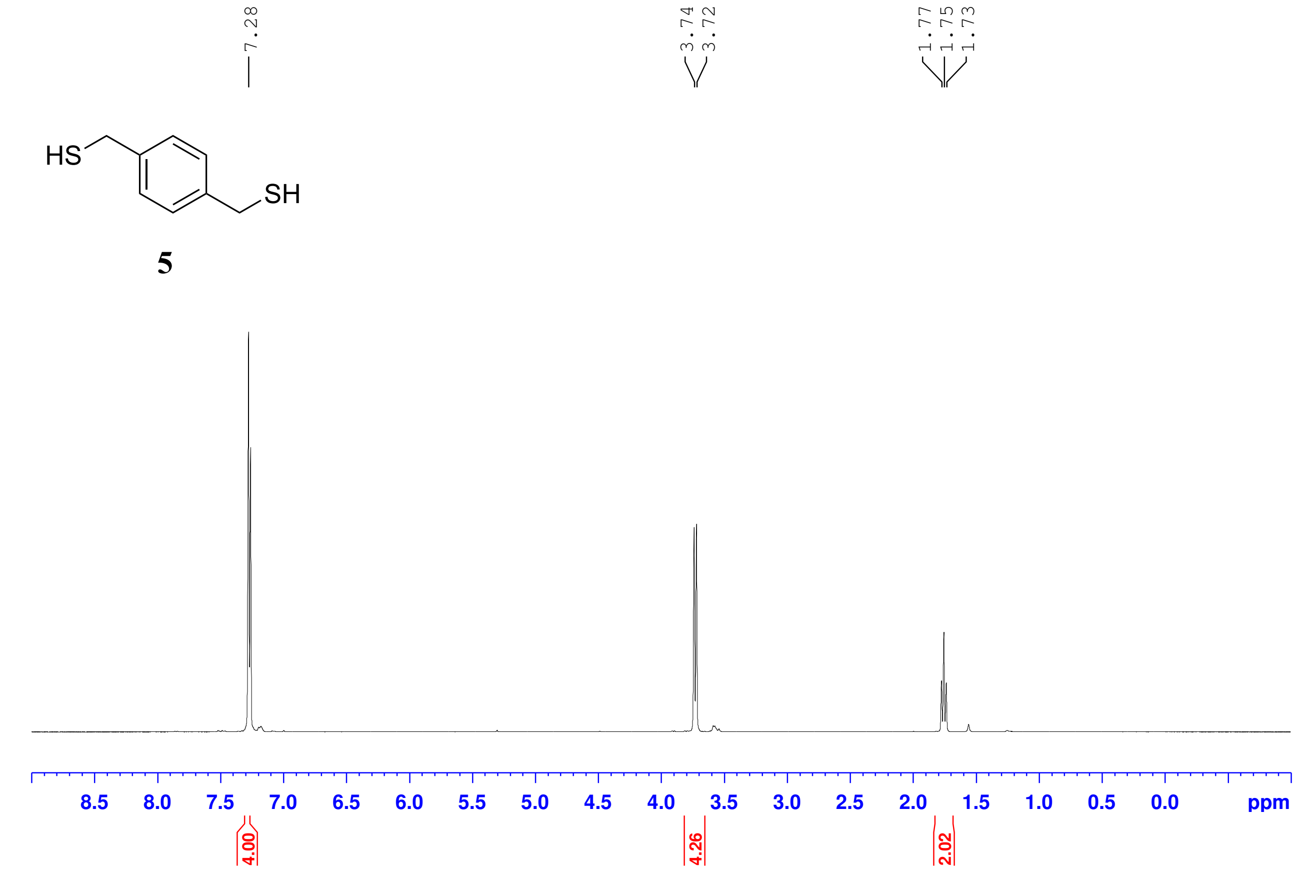

^{1}H NMR of **6** (500 MHz, $CDCl_3$)

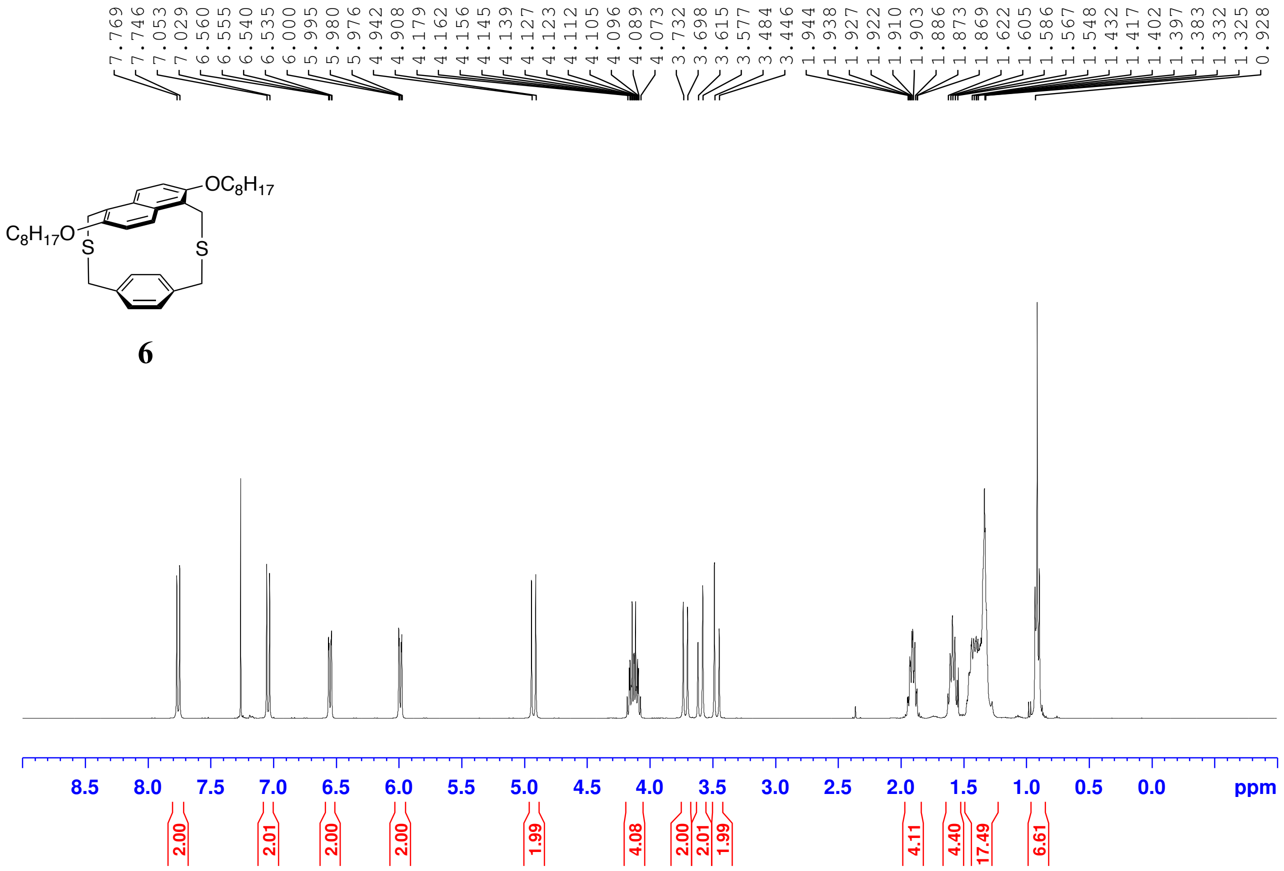

$^{13}C\{^{1}H\}$ NMR of **6** (125 MHz, $CDCl_3$)

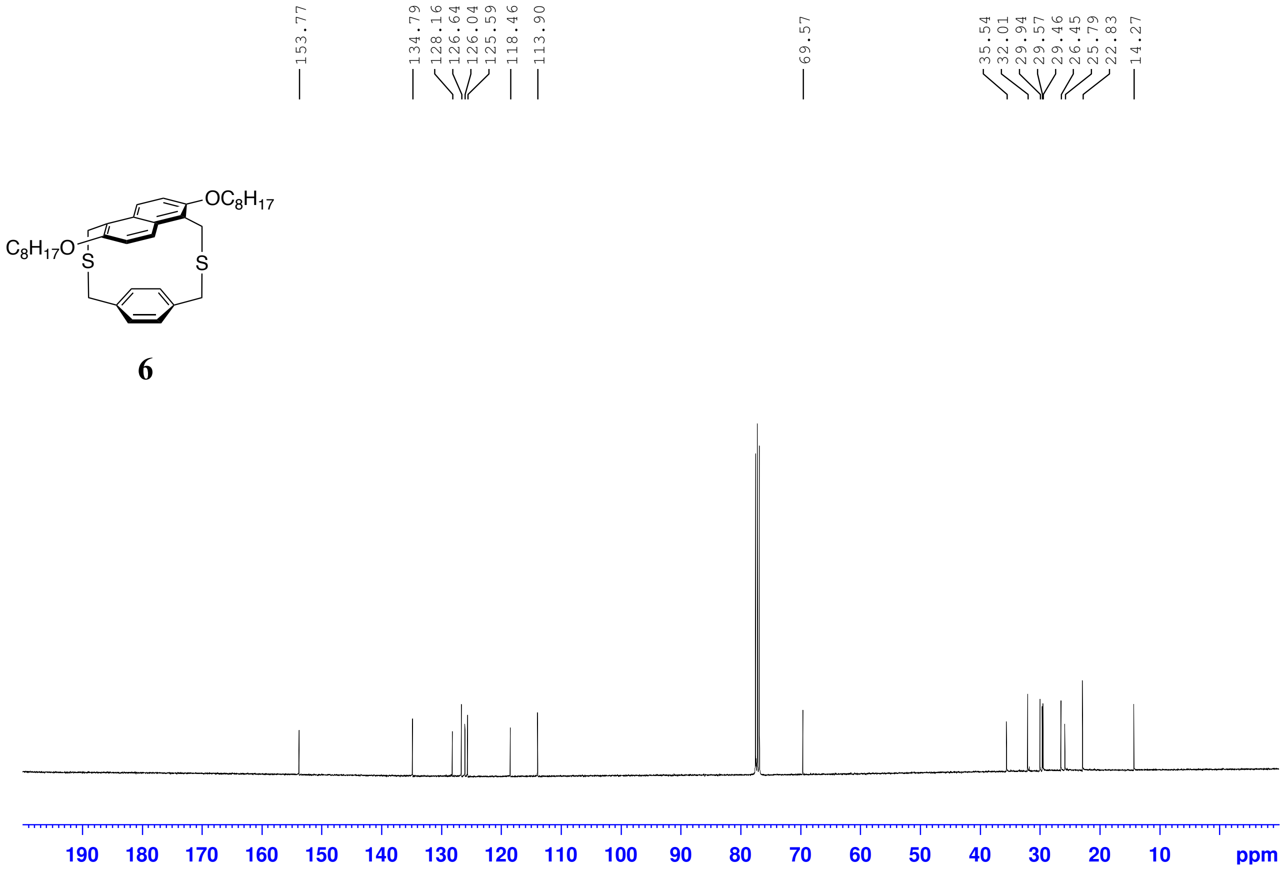

^{1}H NMR of **7** (500 MHz, $CDCl_3$)

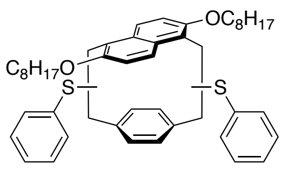

7

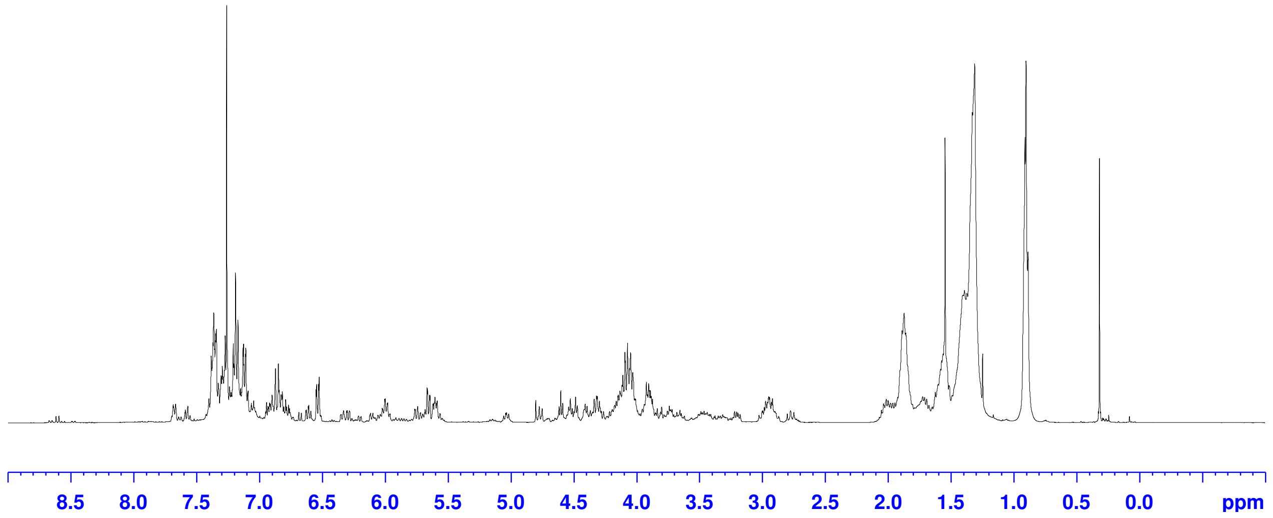

$^{13}C\{^{1}H\}$ NMR of **7** (125 MHz, $CDCl_3$)

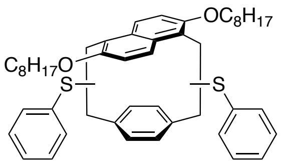

7

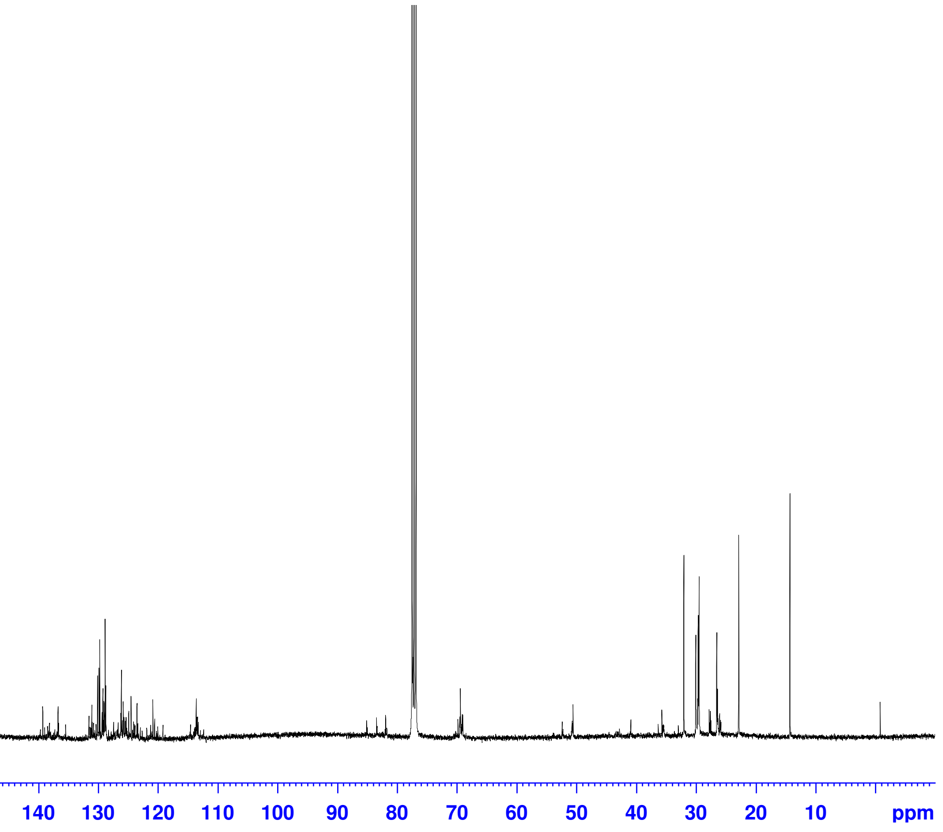

^{1}H NMR of **8** (500 MHz, $CDCl_3$)

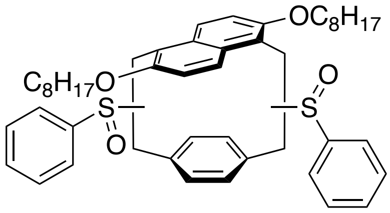

8

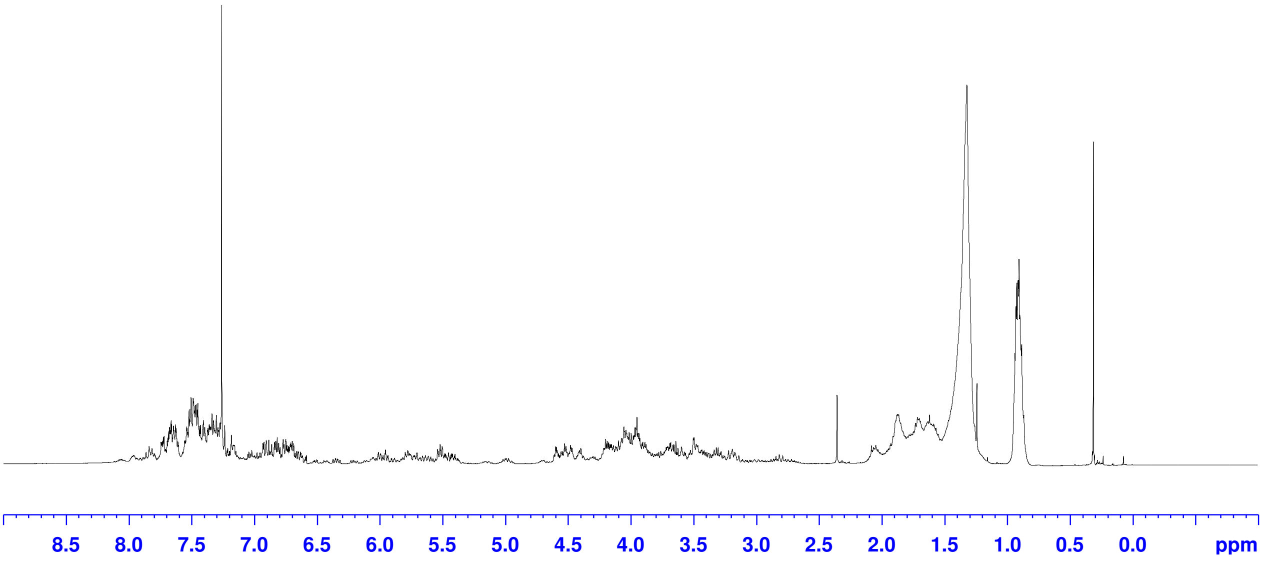

^{13}C{^{1}H} NMR of **8** (125 MHz, $CDCl_3$)

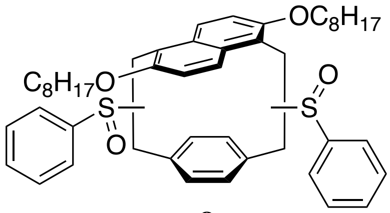

8

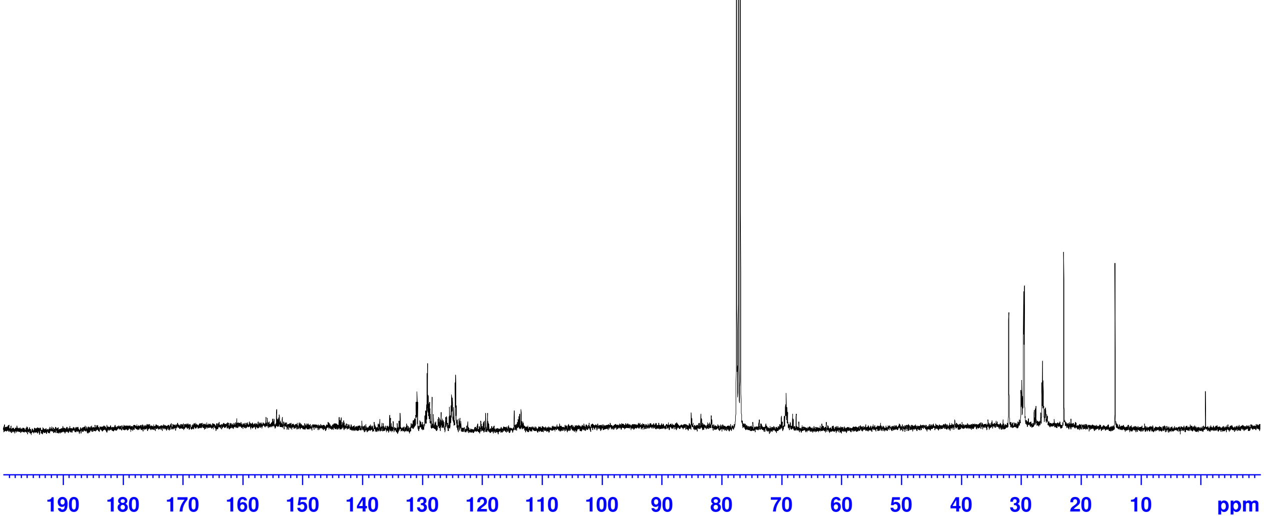

^{1}H NMR of **9** (500 MHz, $CDCl_3$)

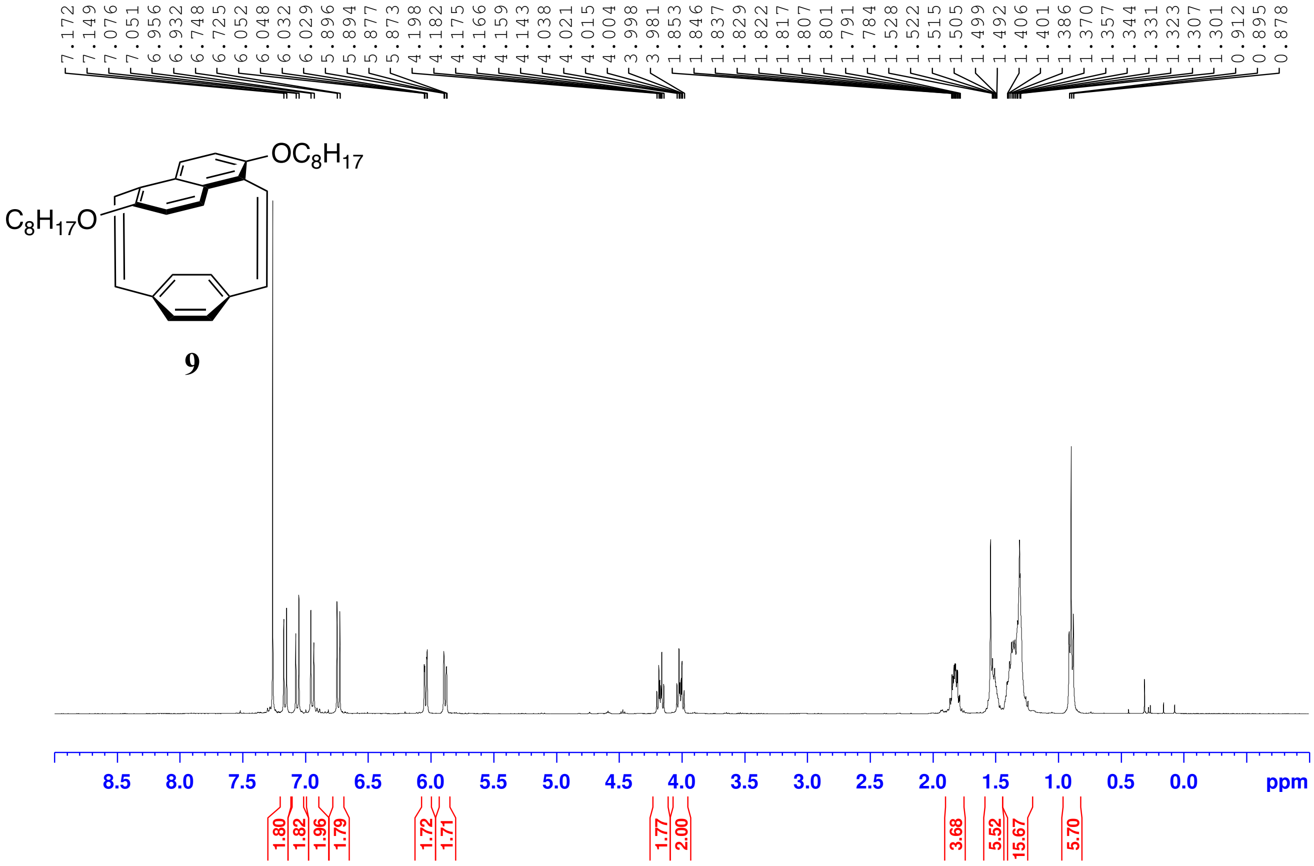

$^{13}C\{^{1}H\}$ NMR of **9** (125 MHz, $CDCl_3$)

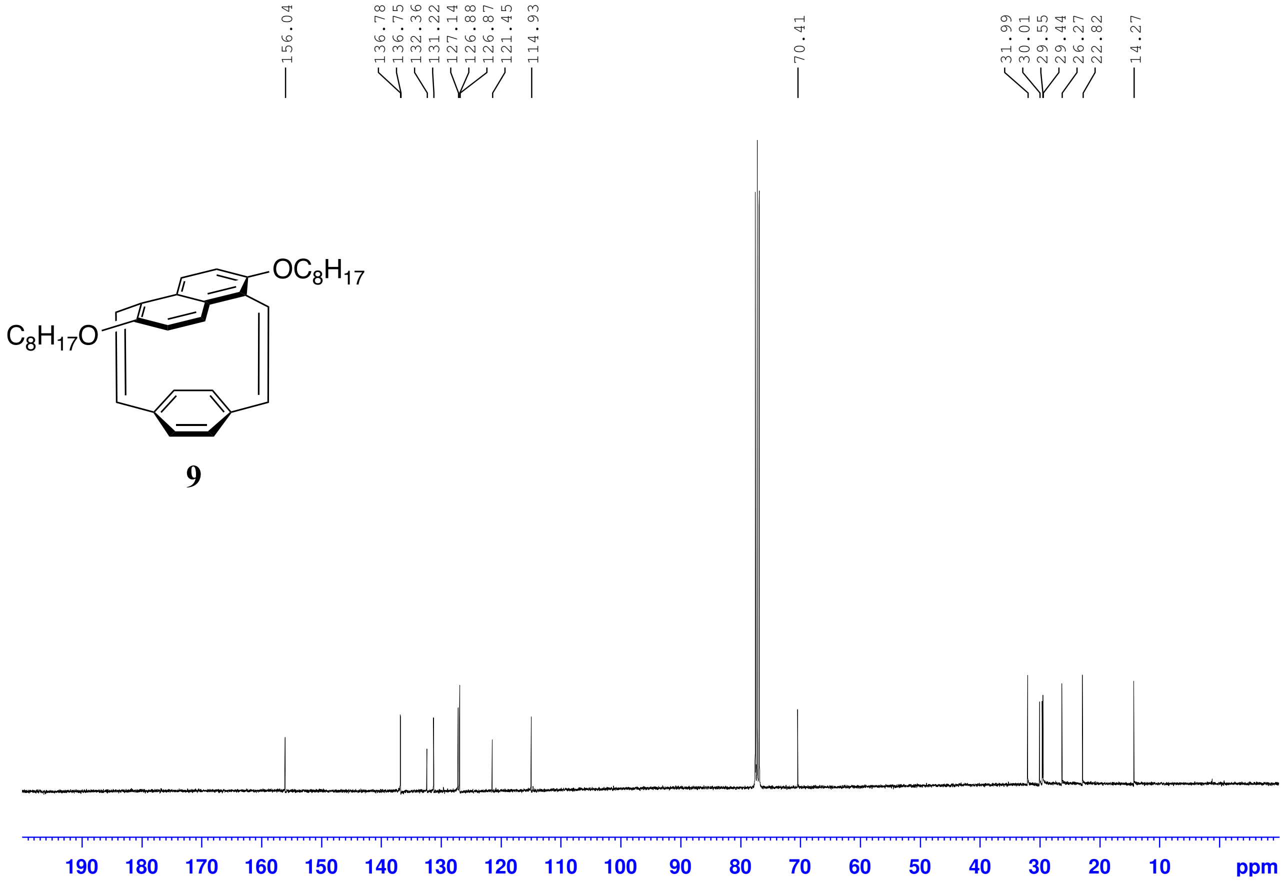

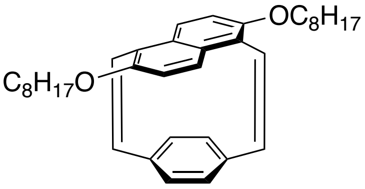

9

HSQC of **9**

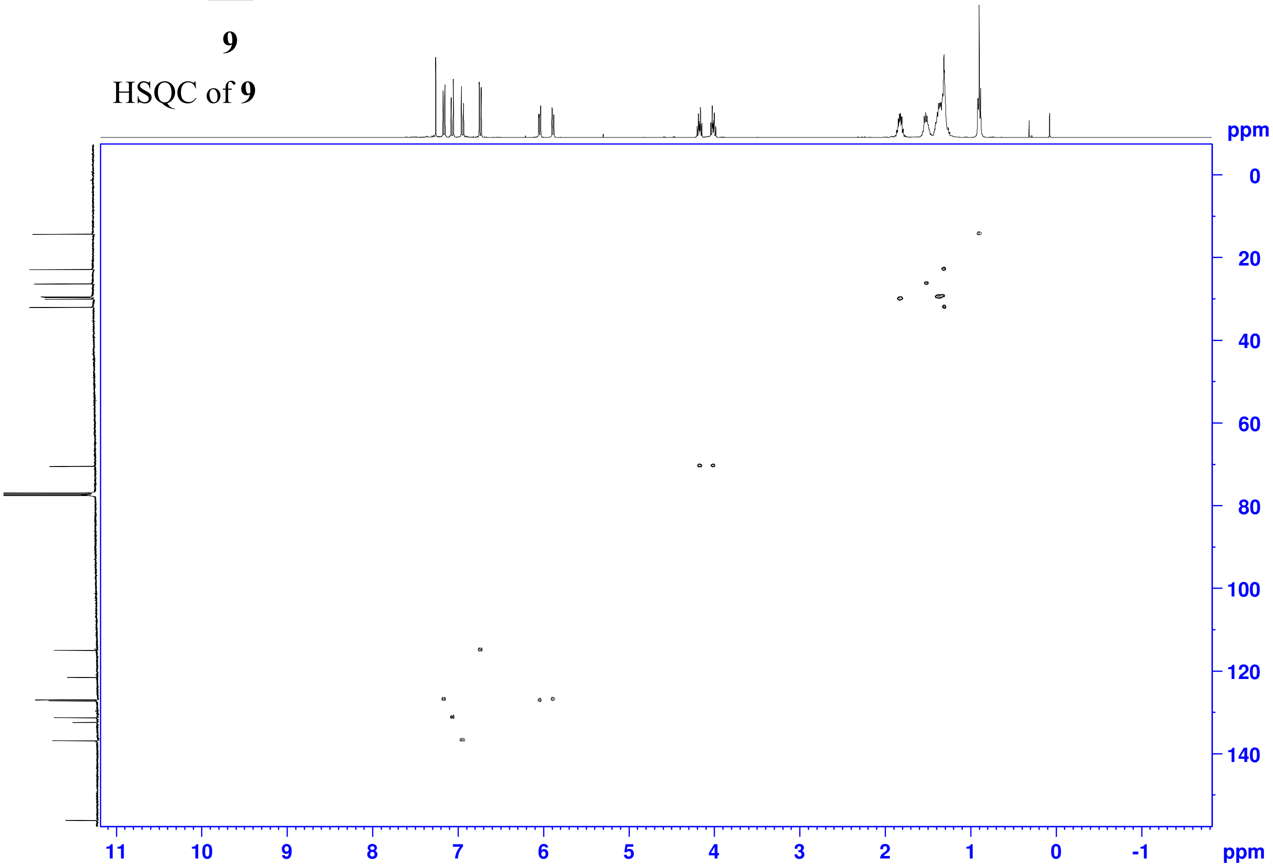

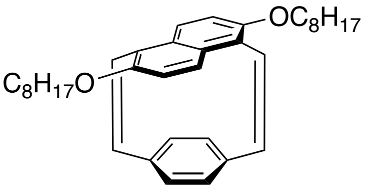

9

HMBC of **9**

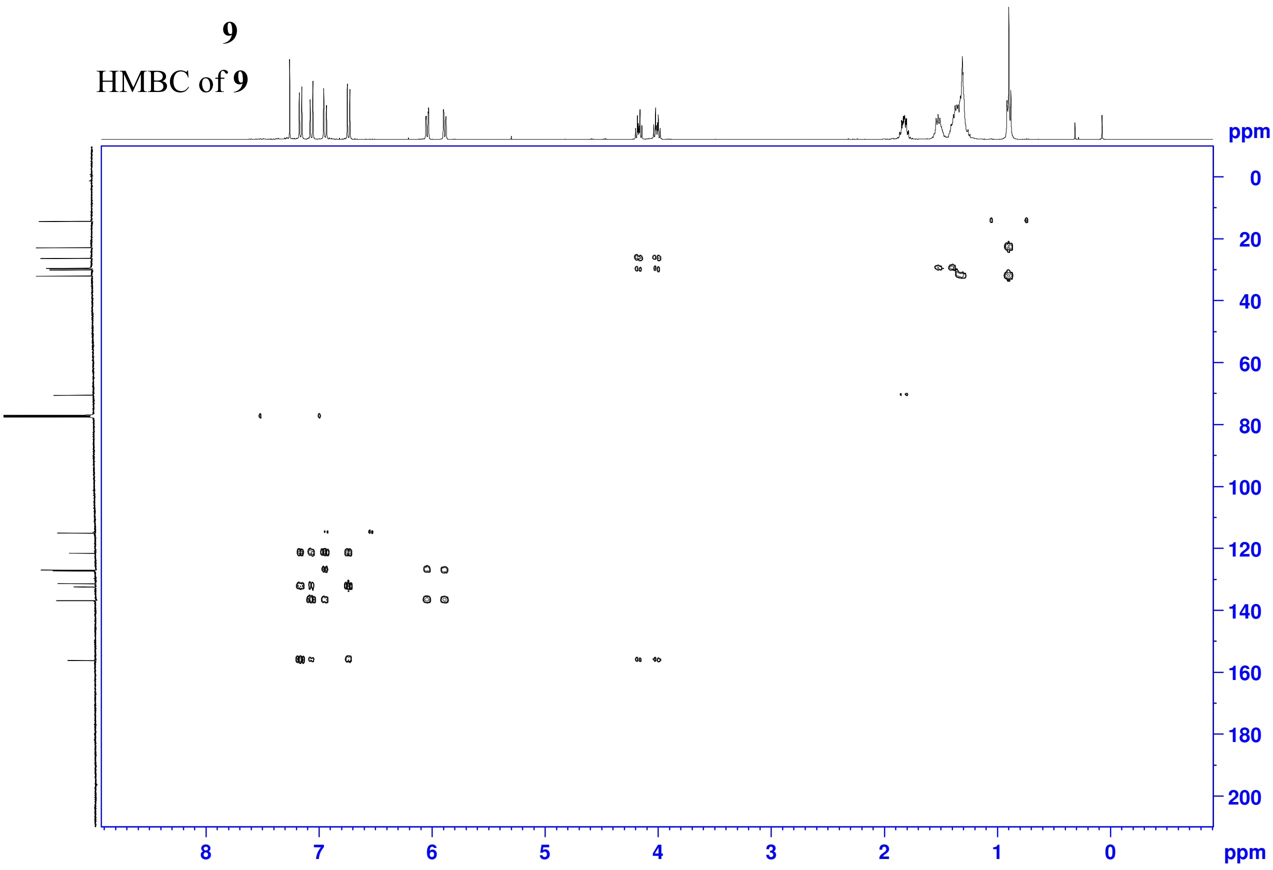

Supplement: Supplementary file 1 — jo3c00880_si_001.pdf [file jo3c00880_si_001.pdf]
